# Genomic landscape of NDM-1 producing multidrug-resistant *Providencia stuartii* causing burn wound infections in Bangladesh

**DOI:** 10.1038/s41598-024-51819-9

**Published:** 2024-01-26

**Authors:** Spencer Mark Mondol, Israt Islam, Md. Rafiul Islam, Shahriar Kabir Shakil, Nadira Naznin Rakhi, Jannatul Ferdous Mustary, Amiruzzaman, Donald James Gomes, Hussain Md. Shahjalal, Md. Mizanur Rahaman

**Affiliations:** 1 Department of Microbiology, University of Dhaka, Dhaka, 1000 Bangladesh; 2 Microbiology Department, Sheikh Hasina National Institute of Burn and Plastic Surgery, Dhaka, 1000 Bangladesh; 3 Department of Medicine, Sir Salimullah Medical College, Dhaka, 1000 Bangladesh; 4 Department of Biochemistry and Molecular Biology, Jahangirnagar University, Savar, Dhaka, 1342 Bangladesh; 5 Department of Biotechnology and Genetic Engineering, Noakhali Science and Technology University, Noakhali, 3814 Bangladesh

**Keywords:** Computational biology and bioinformatics, Genome informatics, Microbiology, Bacterial genomics

## Abstract

The increasing antimicrobial resistance in *Providencia stuartii* (*P. stuartii*) worldwide, particularly concerning for immunocompromised and burn patients, has raised concern in Bangladesh, where the significance of this infectious opportunistic pathogen had been previously overlooked, prompting a need for investigation**.** The two strains of *P. stuartii* (*P. stuartii* SHNIBPS63 *and P. stuartii* SHNIBPS71) isolated from wound swab of two critically injured burn patients were found to be multidrug-resistant and *P. stuartii* SHNIBPS63 showed resistance to all the 22 antibiotics tested as well as revealed the co-existence of *bla*_VEB-6_ (Class A), *bla*_NDM-1_ (Class B), *bla*_OXA-10_ (Class D) beta lactamase genes. Complete resistance to carbapenems through the production of NDM-1, is indicative of an alarming situation as carbapenems are considered to be the last line antibiotic to combat this pathogen. Both isolates displayed strong biofilm-forming abilities and exhibited resistance to copper, zinc, and iron, in addition to carrying multiple genes associated with metal resistance and the formation of biofilms. The study also encompassed a pangenome analysis utilizing a dataset of eighty-six publicly available *P. stuartii* genomes (n = 86), revealing evidence of an open or expanding pangenome for *P. stuartii*. Also, an extensive genome-wide analysis of all the *P. stuartii* genomes revealed a concerning global prevalence of diverse antimicrobial resistance genes, with a particular alarm raised over the abundance of carbapenem resistance gene *bla*_NDM-1_. Additionally, this study highlighted the notable genetic diversity within *P. stuartii*, significant informations about phylogenomic relationships and ancestry, as well as potential for cross-species transmission, raising important implications for public health and microbial adaptation across different environments.

## Introduction

Antimicrobial resistance of bacteria is a phenomenon that had been at the peak of utmost concern, notably in the field of infectious diseases since last two decades^1^. Many of the advances in the design and development of antibiotics and antimicrobial drugs have resulted from the extensive effort and meticulous action to combat the ever-evolving mechanisms of resistance that render most of the existing antibiotics obsolete, thus prompting the investigation for introducing new-found or modified molecules having potentiality to be more effective and more resilient^2^. Burns are one of the most common and devastating form of trauma and injury^3^. On a global scale, burns constitute highly severe injuries, contributing to over 265,000 fatalities worldwide^4^. In the specific context of Bangladesh, an annual toll of approximately 3000 individuals succumb to injuries attributable to burns^5^. A cross-sectional survey, conducted from January to December 2003 on a population basis in Bangladesh, revealed an elevated burn mortality rate among females. The majority of fatalities resulted from accidental incidents, with a mere 5% attributed to self-inflicted burns. Furthermore, the mortality rate was notably higher in rural areas as opposed to urban areas^4^. A research investigation conducted at the National Institute of Burn and Plastic Surgery in Dhaka, Bangladesh, unveiled that for non-lethal burn incidents, demographic groups at elevated risk encompassed young adult males in their early thirties belonging to lower socioeconomic strata^5^. Within the pediatric population, children under eight years old emerged as the most susceptible subgroup. Prevalent non-lethal burn categories included flame-induced injuries (35%), electrical burns (31%), and scald injuries (24%). Regarding mortality cases, high-risk demographics comprised young adult males and children approximately eight years of age. The average extent of total body surface area (TBSA) affected in these instances stood at 46.4%, with complications arising from flame burns constituting the cause of death in 65% of cases^5^. Microbial infection in severe burn wounds by Multidrug-Resistant (MDR) organisms is a serious matter of concern and is a leading cause of morbidity and mortality in hospitalized burn patients^6^. A number of organisms are responsible for causing infections in the wounds of burn patients including MDR Enterobacteriaceae being a major concern. Among the members of Enterobacteriaceae family causing severe infections in burn patients, *Providencia stuartii* (*P. stuartii*) is one of the most alarming and matter of growing concern because of its intrinsic resistance to several antibiotics including colistin and gradual occurrence to complete resistance toward carbapenems, considered to be the last line antibiotics against this particular infectious agent^7^. To date, there has been limited documentation of *P. stuartii* in Bangladesh, particularly in the context of burn wound infections. Some previously published reports from Bangladesh consistently highlighted *Pseudomonas aeruginosa (P. aeruginosa) * as the predominant microorganism associated with infections in burn patients^8–10^. Subsequent to the prevalence of *P. aeruginosa*, other identified pathogens in burn wound infections included *Staphylococcus aureus*, *Proteus* spp., *Klebsiella* spp., *Acinetobacter* spp., and *Escherichia coli*, as documented in the scientific literatures^8–11^. *Providencia* spp. belong to the Morganellaceae family of the order Enterobacterales^12^. *P. stuartii* is a gram-negative rod-shaped bacterium commonly found in soil, water and sewage. It is the most common *Providencia* species capable of causing human infections and is an opportunistic pathogen. *P. stuartii* is well known and characterized for causing nosocomial infections which puts immune compromised elderly individuals at a greater risk^13^. Occurrence of sepsis because of *P. stuartii* infection is considered to be a new challenge in the treatment of thermal injury. In addition to that, many studies have established that, *P. stuartii* has the ability to migrate to several organs causing endocarditis, peritonitis, pericarditis and meningitis^13^. The extensive and alarming antimicrobial resistance pattern and its severe invasive properties along with uprising infection and mortality rate, makes *P. stuartii* indisputably a potential threat and to be considered a matter of great concern as well as a significant, cabalistic and interesting subject to research in order to investigate its pathogenicity, antimicrobial resistance mechanisms, alternative and effective treatment methods.

Carbapenems, the most potent class of β-lactams which are the efficacious addition in our antibiotic armamentarium because of its broad spectrum activity against both Gram-positive organisms and Gram negative especially multidrug-resistant Gram-negative bacilli^14^. Association of mobile genetic elements (MGEs) with carbapenemases even crossed the species barrier resulting in the worldwide rapid transmission of carbapenemases in nonclonally related isolates and species and the most important types based on rapid and extensive epidemiological dissemination and clinical relevance are *bla*_KPC_-type, *bla*_OXA_-type, *bla*_VIM_-type, *bla*_NDM_-type of cabapenemases^15^. As *P. stuartii* is intrinsically resistant to many antibiotics such as Gentamycin, Tobramycin, Ampicillin, Cefazolin including Colistin; the carbapenems are considered to be the last line drug to treat infections caused by this organism^16^. Carbapenem resistant *P. stuartii* are increasingly reported mainly due to *bla*_NDM-1_^17^. So, emerging resistance of this organism to carbapenems could be undoubtedly alarming also in the perspective of Bangladesh as worldwide. Whole genome sequencing (WGS), also known as complete genome sequencing, is the process of determining the entire or nearly the entire DNA sequence of an organism’s genome at a single time which reveals almost every detail encoded about that particular organism^18^. The recent advancement in bacterial whole genome sequencing approach made it a widely used technique in research, clinical diagnostic and public health laboratories. It enables high resolution characterization of bacterial pathogens in terms of properties that include antibiotic resistance, molecular epidemiology, and virulence^19^.

In the perspect of Bangladesh, *P. stuartii* is not advertently reported and no report has been drawn up or published yet regarding the occurrence of this potential threat in burn patients where its role is certainly serious. In addition to the lack of report about the investigation of *P. stuartii* in Bangladesh, no whole genome sequencing data of this particular potential threatening organism has been published yet from Bangladesh. The unaware inattention toward this infectious opportunistic pathogen which is being reported as highly dangerous for burn patients worldwide, roused the importance and concernment to investigate about this pathogen in Bangladesh, most importantly in burn and immunocompromised patients. In this study, we have reported about the cultural and genomic characteristics of two MDR isolate *Providencia stuartii* SHNIBPS63 (Carbapenem resistant) and *Providencia stuartii* SHNIBPS71 (Carbapenem sensitive), isolated from infected burn wound of two critically injured burn patient.

## Results and discussion

### Whole genome sequence analysis, identification and mapping

**Figure 1:**
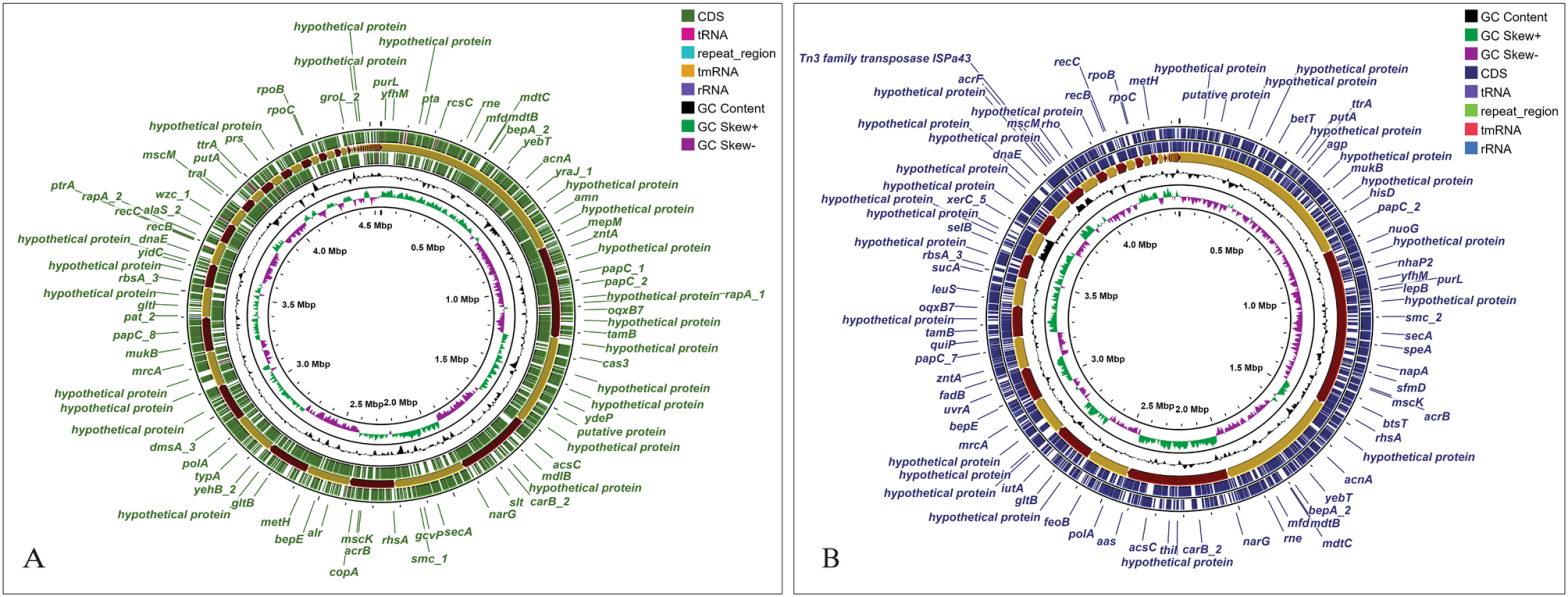
Genome maps of the analyzed genomes. (**A**) *P. stuartii* SHNIBPS63, (**B**) *P. stuartii* SHNIBPS71.

The identification through KmerFinder predicted the two isolates to be *Providencia stuartii (P. stuartii*). Besides, the identification through Average Nucleotide Identity (ANI) approach validated the identification and revealed the test isolates to be *Providencia stuartii* as the test isolates showed significant average nucleotide identity of more than 99% with the references (Table 1). Along with that, the isolates were identified as *P. stuartii* by 16S rDNA-based identification as well. Following the identification, the two test isolates were designated as *Providencia stuartii* SHNIBPS63 and *Providencia stuartii* SHNIBPS71. The draft genome analysis revealed that the genomes were of quite quality having around 100 percent completeness and less than 0.6% contamination which indicated toward the good quality and consistency of the genome. The genome size and GC contents of the sequenced isolates were consistent with the published genomes of *P. stuartii*. Detailed annotation of the two isolates can be found in Table 2**.** The genome maps revealed constructive and diverse features of both genome (Fig. 1). The sources of each contigs were listed in Table 3.

**Table 1:** Identification of the test isolates.

(a) Average nucleotide identity (ANI)				
Isolate	Reference	ANI (%)		
*Providencia stuartii* SHNIBPS63	*Providencia stuartii* MRSN 2154	99.95		
	*Providencia stuartii* ATCC 33672	99.48		
*Providencia stuartii* SHNIBPS71	*Providencia stuartii* MRSN 2154	99.47		
	*Providencia stuartii* ATCC 33672	99.49		

(b) BLASTn results				
Test isolate	Matched isolate	Query cover (%)	Percent identity (%)	e value
*Providencia stuartii* SHNIBPS63	*Providencia stuartii* FDAARGOS_645	100	100	0.0
	*Providencia stuartii* MF1	100	100	0.0
*Providencia stuartii* SHNIBPS71	*Providencia stuartii* FDAARGOS_645	100	100	0.0
	*Providencia stuartii* AR_0026	100	100	0.0

**Table 2:** General features of the annotated genomes.

BV-BRC (RASTtk) annotation	*Providencia stuartii SHNIBPS63*	*Providencia stuartii SHNIBPS71*	Prokka annotation	*Providencia stuartii SHNIBPS63*	*Providencia stuartii SHNIBPS71*
Completeness of genome	100	100	Contigs	82	54
Contamination	0.1	0.5	Bases	4,540,408	4,428,595
Contigs	82	54	CDS	4240	4083
Genome length	4,540,408	4,428,595	rRNA	5	5
GC content	41.346664	41.665268	Repeat Region	3	5
Contig L50	6	4	tRNA	58	59
Contig N50	185,505	439,553	tmRNA	1	1
CDS	4555	4378			
Hypothetical protein	1153	1067			
% features that are hypothetical	35.02	34.19			
tRNA	61	63			
rRNA	7	5

**Table 3:** Source of each node detected in the draft genomes.

*P. stuartii* SHNIBPS63		*P. stuartii* SHNIBPS71	
Source	Node no.	Source	Node no.
Chromosome	1–17, 19–32, 35–36, 39, 41, 43–44, 46, 49–53, 55–57, 59–60, 64, 66–68, 71–72, 75–76, 80	Chromosome	1–12, 14–15, 17–25, 27, 29–31, 33, 35–36, 39, 50
Plasmid	18, 33, 38, 42, 45, 63	Plasmid	13, 16, 26, 28, 32, 34, 42, 44–46, 49
Phage	34, 37, 40, 47–48, 54, 58, 61–62, 65, 69–70, 73–74, 77–79, 81–82	Phage	37–38, 40–41, 43, 47–48, 51–54

The draft genome sequences of the two isolate *Providencia stuartii* SHNIBPS63 and *Providencia stuartii* SHNIBPS71 were deposited in DDBJ/ENA/GenBank of NCBI under the accession number JASDEX000000000 and JASDEW000000000 respectively. From the annotated data, it was found that both strains contained > 2000 genes in common. Around 35% hypothetical proteins (HP) are indicative toward the functional diversity, evolutionary divergence along with the possibility to contain novel genes or proteins having potential significance. By definition, an HP is a predicted product derived from an open reading frame (ORF) whose translation has not been experimentally demonstrated, and its functional significance remains to be characterized^20^. From extensive analysis of these hypothetical proteins from *P. stuartii*, it might be possible to find out proteins having novel functions, new diagnostic markers for *P. stuartii* or drug targets to combat this pathogenic bacterium. The genome size and GC contents of the sequenced isolates were consistent with the published reference genomes of *P. stuartii*.

### Antimicrobial susceptibility testing and cultural characterization

The two targeted isolates showed multidrug resistance pattern which is depicted in Supplementary Fig. 1. The *P. stuartii* SHNIBPS63 showed resistance to all the 22 antibiotics tested against it. The other isolate *P. stuartii* SHNIBPS71 exerted sensitivity to meropenem, doripenem and ciprofloxacin. It showed intermediate sensitivity to imipenem, tigecycline, aztreonam and also showed tolerance against imipenem after the tolerance assay performed against carbapenems. As carbapenems, polymyxins and according to some report tigecycline is considered to be the last line or resort of antibiotics^21^, CRE (Carbapenem Resistant Enterobacterales) like *P. stuartii* SHNIBPS63 is a matter of concern as *P. stuartii* is known to be naturally resistant in colistin and tigecycline. Only *P. stuartii* SHNIBPS63 gave positive result in Modified Hodge test which indicated toward the production of carbapenemase. Along with that, both isolates were predicted to be strong biofilm former. The ability of bacteria to form biofilm is one the mechanism of resistance to survive in the presence of antibiotics^22^. Metal sensitivity test revealed both isolates to be resistant in 0.01 M iron, copper and zinc solution. The isolates gave characteristic yellow/orange colonies on MacConkey agar and pinpoint colorless colonies on Cetrimide agar media. They were able to ferment glucose, but not lactose and sucrose. Additionally, they showed characteristics such as indole production, nitrate reduction, a positive methyl red reaction, and were oxidase negative but catalase positive. These traits matched the cultural characteristics typically observed in *P. stuartii*.

### Antimicrobial resistance genes and mobile genetic elements investigation

**Figure 2:**
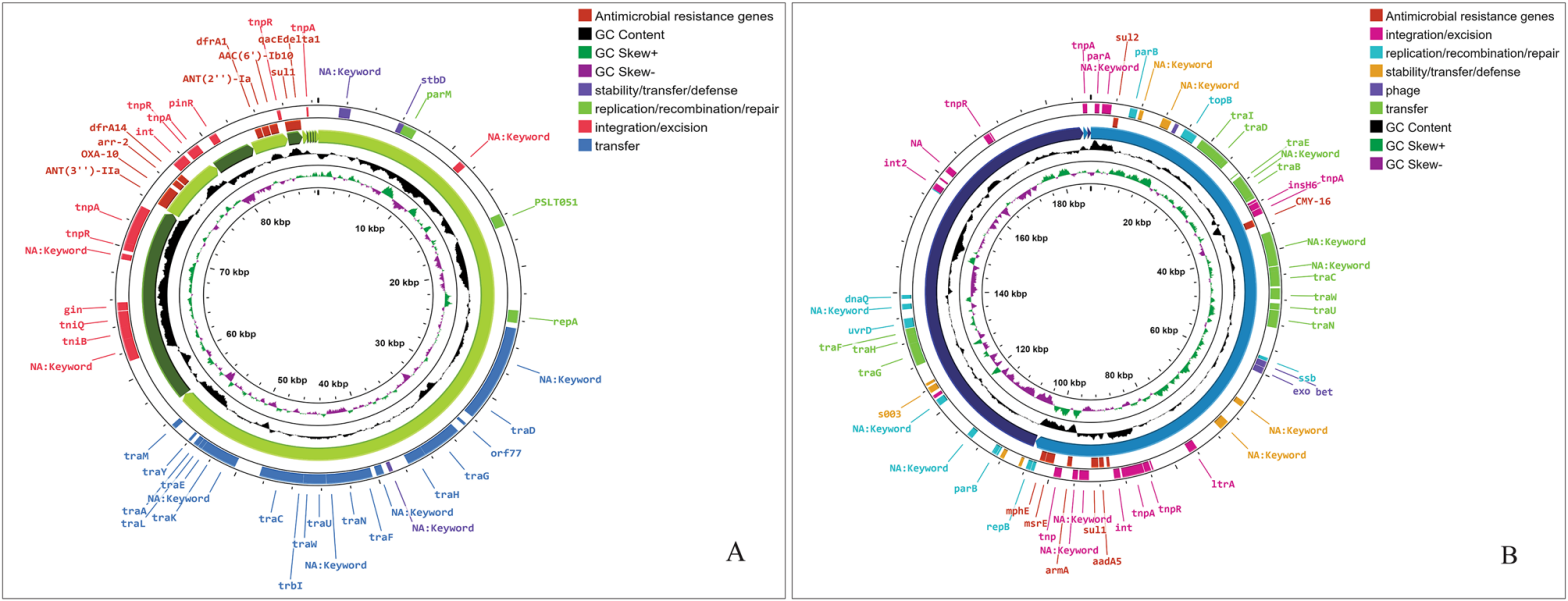
Plasmid mapping and annotation showing antimicrobial resistance genes carried by the plasmids and genes associated with their dissemination. (**A**) Plasmid mapping of *Providencia stuartii* SHNIBPS63. (**B**) Plasmid mapping of *Providencia stuartii* SHNIBPS71.

**Figure 3:**
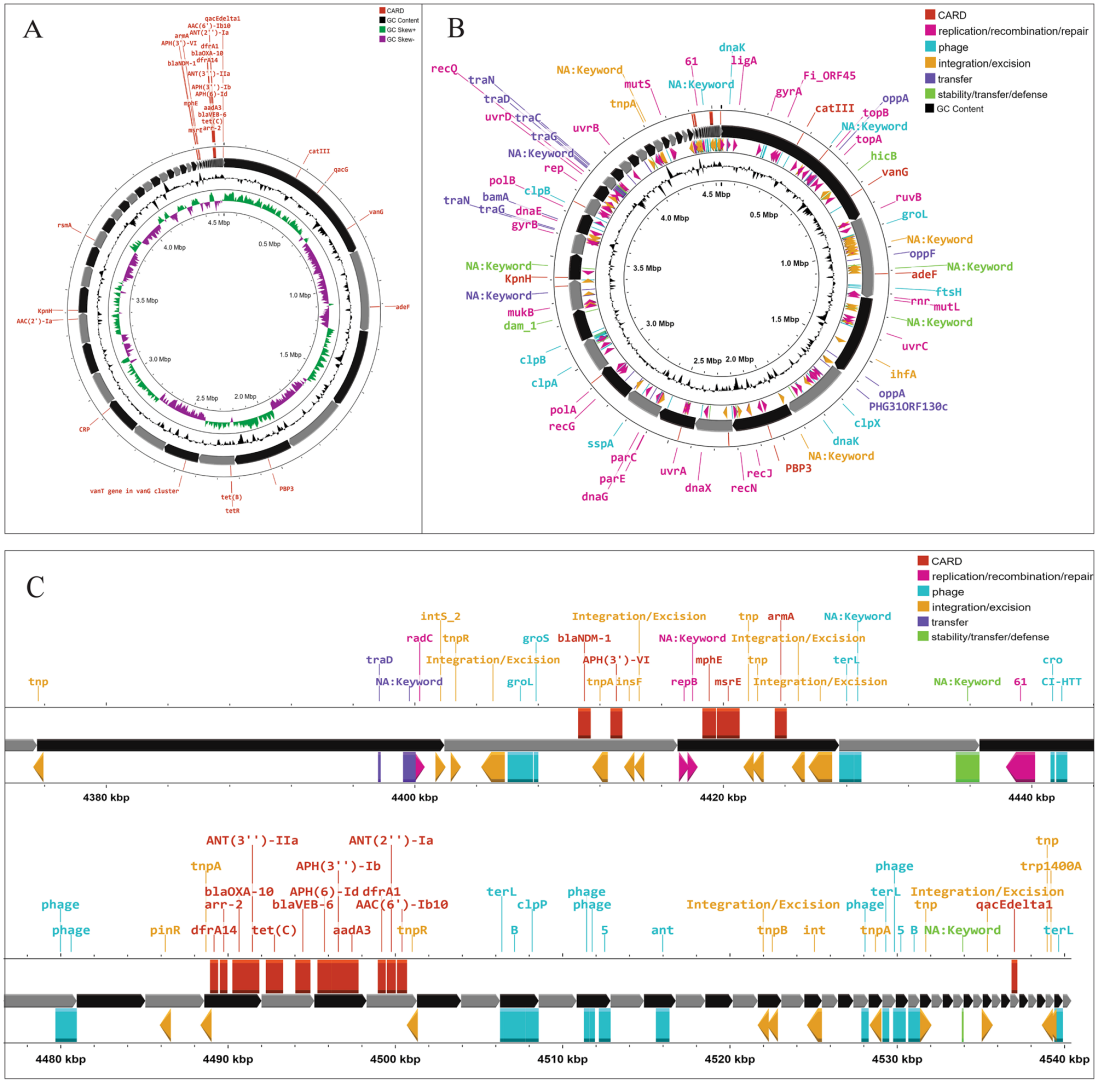
Mapping of antimicrobial resistance genes and mobile genetic elements in carbapenem resistant *Providencia stuartii* SHNIBPS63. (**A**) Antimicrobial resistance genes mapping. (**B**) The organization of mobile genetic elements in the genome. (**C**) Organization and mapping of antimicrobial resistance genes with surrounding mobile genetic elements.

The antimicrobial resistance genes investigation revealed that *P. stuartii* SHNIBPS63 harboured plethora of genes which are responsible for conferring resistance to many antibiotics. This particular isolate is co-harboured with *bla*_VEB-6_ (Class A), *bla*_NDM-1_ (Class B) and *bla*_OXA-10_ (Class D) which confer resistance to beta lactams. The localization and source investigation of the antimicrobial resistance genes revealed that *bla*_NDM-1_ and *bla*_VEB-6_ genes were predicted to be located in the chromosome. NDM-1, the product of *bla*_NDM-1,_ which is a metallo beta lactamase enzyme (carbapenemase), has the capability to confer resistance against carbapenems. According to plasmid mapping, it was determined that 11 antimicrobial resistance genes, including *bla*_OXA-10_, were likely carried by plasmids (Fig. 2). Conversely, in the case of *P. stuartii* SHNIBPS71, it possessed the plasmid-mediated class C beta-lactamase gene, *bla*_CMY-16_. The presence of *bla*_CMY-16_ gene in *P. stuartii* SHNIBPS71 conferred resistance to certain beta-lactams, notably cephalosporins, but not able to protect the bacteria from carbapenems. No other beta lactam resistance genes were present in this isolate which indicated toward the sensitivity of this isolate to carbapenems. All the antimicrobial resistance genes carried by the two isolates are listed in Table 4. Total nine antimicrobial resistance genes were anticipated to be plasmid mediated in this isolate. In case of *P. stuartii* SHNIBPS63, the presence of *bla*_NDM-1_ is responsible for conferring complete resistance to carbapenems like meropenem, imipenem, ertapenem and doripenem. Usually, *bla*_NDM-1_ gene is mediated by plasmids and reported to be carried by plasmids^23^. But, in case of *P. stuartii* SHNIBPS63 it was carried in the chromosome which was verified through plasmid and genome analysis. In depth analysis revealed that the *bla*_NDM-1_ gene was surrounded by several insertion sequences, transposase, integrase genes which denoted toward the possibility of the gene to be acquired or inserted into the chromosome through horizontal gene transfer (Fig. 3).

**Table 4:** Investigation of antimicrobial resistance genes.

*Providencia stuartii* SHNIBPS63			*Providencia stuartii* SHNIBPS71		
Class/type	Genes	Antimicrobial agent	Class/type	Genes	Antimicrobial agent
Aminoglycoside	*aac*(6’)-lb	Amikacin, Tobramycin	Aminoglycoside	*aac*(2’)-la	Gentamycin, Tobramycin, Dibekacin, Netilmicin
	*aph*(3’)-VI	Amikacin		*arm*A	Amikacin, Gentamycin, Tobramycin, Netilmicin, Isepamicin
	*aad*A1, *aad*A2b	Spectinomycin, Streptomycin		*aad*A5	Spectinomycin, Streptomycin
	*ant*(2”)-la	Gentamycin, Tobramycin			
	*aac*(2’)-la	Gentamycin, Tobramycin, Dibekacin, Netilmicin			
	*arm*A	Amikacin, Gentamycin, Tobramycin, Netilmicin, Isepamicin			
	*aph*(6)-Id, *aph*(3”)-Ib	Streptomycin			
	*aac*(6’)-lb-cr	Ciprofloxacin			
Tetracycline	*tet*(A)	Tetracycline antibiotics	Tetracycline	*tet*(B), *tet*R	Tetracycline antibiotics
	*tet*(B), *tet*R	Tetracycline antibiotics			
Beta-lactam	*bla*_NDM-1_	Amoxicillin, Amoxicillin + Clavulanic acid, Ampicillin, Ampicillin + Clavulanic acid, Cefepime, Cefixime, Cefotaxime, Cefoxitin, Ceftazidime, Ertapenem, Imipenem, Meropenem, Piperacillin, Piperacillin + Tazobactam, Temocillin	Beta-lactam	*bla*_CMY-16_	Amoxicillin, Amoxicillin + Clavulanic acid, Ampicillin, Ampicillin + Clavulanic acid, Cefotaxime, Cefoxitin, Ceftazidime, Piperacillin, Piperacillin + Tazobactam, Ticarcillin, Ticarcillin + Clavulanic acid
	bla_OXA-10_	Amoxicillin, Ampicillin, Aztreonam, Piperacillin, Piperacillin + Tazobactam			
	bla_VEB-6_	Monobactam, Cephalosporin			
Quinolone	*qnr*D1, *aac*(6’)-lb-cr	Ciprofloxacin	Quinolone	*qnr*D1	Ciprofloxacin
StreptograminB	*msr*(E)	Erythromycin,Azithromycin,Quinupristin,Pristinamycin	StreptograminB	*msr*(E)	Erythromycin, Azithromycin, Quinupristin, Pristinamycin
Folate pathway antagonist	*sul*1	Sulfamethoxazole	Folate pathway antagonist	*sul*2	Sulfamethoxazole
	*dfr*A1, *dfr*A14	Trimethoprim		*dfr*A1	Trimethoprim
Aminocyclitol	*aad*A2b, *aad*A1	Spectinomycin, Streptomycin	Aminocyclitol	*aad*A5	Spectinomycin, Streptomycin
Rifamycin	*arr*-2	Rifampicin			
Macrolide	*mph*(E)	Erythromycin	Macrolide	*mph*(E)	Erythromycin
	*msr*(E)	Erythromycin, Azithromycin, Quinupristin, Pristinamycin		*msr*(E)	Erythromycin, Azithromycin, Quinupristin, Pristinamycin
Amphenicol	*cat*A3	Chloramphenicol	Amphenicol	*cat*A3	Chloramphenicol
Quaternary ammonium compound	*qac*E	Benzylkonium chloride, Ethidium Bromide, Chlorhexidine, Cetylpyridinium chloride	Quaternary Ammonium Compound	*qac*E	Benzylkonium chloride, Ethidium Bromide, Chlorhexidine, Cetylpyridinium chloride

**Figure 4:**
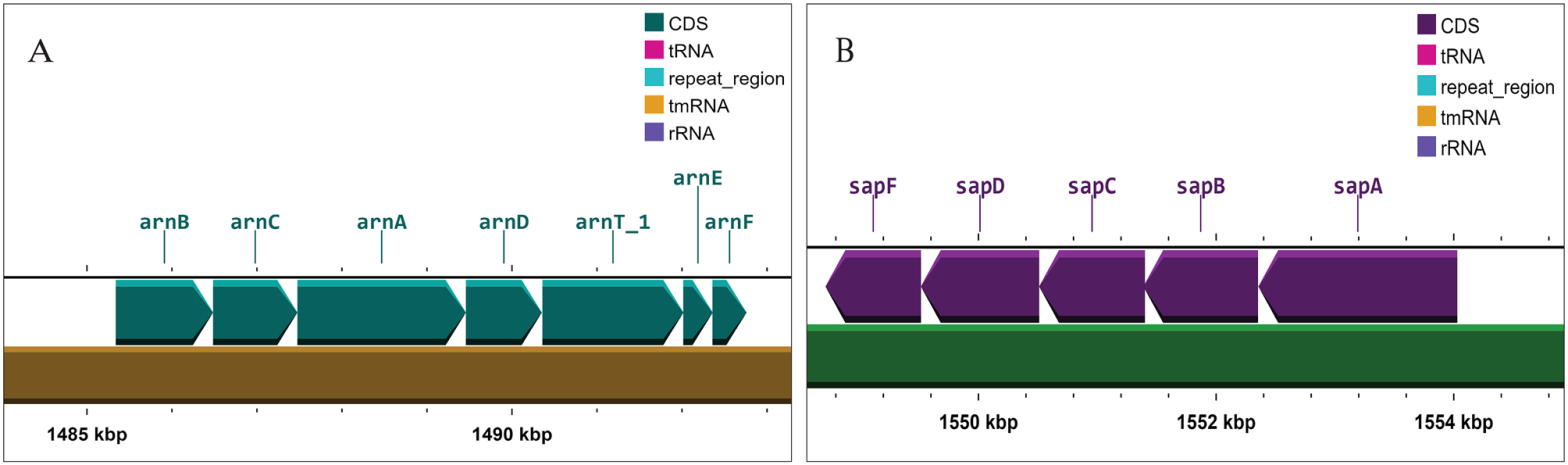
Presence of *arn* and *sap* operon in the genome of *P. stuartii* SHNIBPS63. (**A**) Genomic organization of the *arn*BCADTEF operon. (**B**) Genomic organization of the *sap*ABCDF operon.

Investigation on colistin resistance in both test isolates revealed the presence of *arn*BCADTEF operon (Fig. 4) which was predicted to be responsible for conferring intrinsic resistance to colistin. The *arn*BCADTEF operon encodes for a set of enzymes that modify lipopolysaccharides (LPS), which are a major component of the outer membrane of gram-negative bacteria. The 4-amino-4-deoxy-l-arabinose (l-Ara4N) chemical groups are specifically added to the lipid A portion of LPS by the *arn*BCADTEF operon^24^. This can inhibit the binding of colistin to the bacterial membrane and hence reduce the drug's effectiveness. Additionally, *sap*ABCDEF operon, *pho*PQ (A two component system), *acr*AB-*Tol*C, *lpx*A/C/D, *emr*A/B/D/E, *ram*A and *ugd* gene sequences were detected having potential role in conferring colistin resistance.

Integron investigation revealed the presence of class I integron in *P. stuartii* SHNIBPS71 and 2 CALIN (Class 3) type and 1 In0 type integron in *P. stuartii* SHNIBPS63. Although studies have indicated that CALIN integrons can contribute to the transmission of resistance to a number of kinds of antibiotics, including aminoglycosides and beta-lactams, their involvement in the dissemination of antibiotic resistance genes is not yet entirely understood^25^. On the other hand, In0-type integrons are of interest because they are thought to represent an intermediate stage in the evolution of active integrons. Some researchers have hypothesized that In0-type integrons might be the precursors of active integrons, and they have the ablility or potentiality to evolve into active integrons by acquiring an integrase gene and associated recombination site^26^. Additionally, several mobile genetic elements were detected in the genomes and especially around the antimicrobial resistance gene cassettes in *P. stuartii* SHNIBPS63 which indicated toward the horizontal transfer of antimicrobial resistance genes (Fig. 3).

### Metal resistance genes and virulence factor genes analysis

Metal resistance genes analysis through BacMet revealed the presence of >100 metal and biocide resistance genes (Supplementary Table 1). The analysis disclosed the presence of genes capable of conferring resistance to several metals such as copper, zinc, iron, cadmium, arsenic, antimony, tungsten and manganese. Both isolates revealed metal and biocide resistance genes such as *acr*A, *acr*B, *env*C, *acr*F, *acr*R, *act*P, *ars*A, *ars*B, *cop*A, *mdt*A, *mdt*B, *mdt*C, *sit*A and many more. The presence of such genes can have important implications in healthcare. For example, in a clinical context, it may signify that the bacterium has developed resistance to metals that are sometimes used in antimicrobial agents. This resistance can make it more challenging to treat infections caused by these bacteria, as traditional antimicrobial treatments may be less effective. The presence of several metal resistance genes in *P. stuartii* infecting the wound of burn patients can limit the use of metal-based therapeutics and medications which are applied in wound treatment and repair. Additionally, the presence of metal resistance genes may suggest that the bacterium has developed a degree of adaptability and resilience, making it potentially more difficult to eradicate. Understanding the presence of these genes can be important for choosing appropriate treatment strategies and managing the spread of drug-resistant bacteria. In a comparative analysis, it was observed that *P. stuartii* SHNIBPS63 possessed *mer*A, *mer*D, *mer*E, *mer*P, and *mer*T genes, which were conspicuously absent in *P. stuartii* SHNIBPS71. These genes play a crucial role in providing resistance against mercury.

PathogenFinder tool predicted the two isolates of *P. stuartii* to be highly pathogenic. Virulence factor genes investigation revealed the presence of a plethora of virulence genes in the two test isolates. Several genes were found having role in adherence and biofilm formation such as *fli*A, *fli*C, *fli*D, *fli*E, *fli*G, *fli*H, *fli*I, *fli*J, *fli*K, *fli*L, *fli*M, *fli*N, *fli*P, *fli*Q, *fli*R, *fli*S, *fli*T, *fli*Z, *flg*K, *flg*H, *flg*F, *flg*D, *flg*B, *flg*M, *flg*N, *rhl*E, *rhl*B, *fim*A, *fim*D, *pil*J, *pil*M, *pil*N, *pil*Q, *pap*C and *pap*D. The abundance of genes related with biofilm formation correlated with the phenotypic trait of being strong biofilm former of the two isolates. Also, virulence genes prediction revealed the presence of *yij*D gene, which has a putative function of invading the brain endothelial cells, is indicative toward the capacity of *P. stuartii* to cause meningitis^27^. Besides, type I, type II, type III, type VI secretion systems and relevant genes were also found in the two isolates. Bacterial secretion systems are critical for the virulence and pathogenicity of various pathogens and in the dynamics of burn wound infection. These systems enable bacteria to deliver toxins, effector proteins, and other molecules into host cells, manipulate host processes, evade the immune system, and acquire essential nutrients^28^. By using secretion systems, bacteria can establish infections, form biofilms, and communicate with each other. Understanding these mechanisms is crucial for combating bacterial infections and developing effective treatments. On the otherhand, chaperone-usher fimbriae and fimbrial clusters were also investigated and numbers of proteins associated with fimbriae formation were detected in our test isolates of *P. stuartii* (Supplementary Table 2). In case of burn wound infection by *P. stuartii*, the role of fimbriae can play pivotal role in case of adherence to host tissues. This adherence is often a crucial initial step in the establishment of infection. Fimbriae facilitate the colonization of host surfaces, allowing pathogens to resist mechanical clearance and evade host immune responses^29^. Some toxins such as actin cross linking toxin VgrG-1, persistence and stress-resistance toxin PasT, toxin-antitoxin system YoeB/YefM were found to be associated with the isolates. Actin cross linking toxins are reported to specifically target phagocytic cells to promote survival of bacteria after the onset of innate immune defenses^30^. Previous investigations have demonstrated the essential role of VgrG-1 in facilitating host cell cytotoxicity through the Type VI Secretion System (T6SS) and in hindering phagocytosis mediated by actin cytoskeleton, thereby preventing bacterial engulfment by macrophages^31^. These mechanisms could potentially exert a substantial influence on the initiation and advancement of burn wound infections caused by *P. stuartii*. Several other virulence factor genes such as *rfa*D, *rfa*C, *wec*A, *gal*E, *gal*U, *bio*A, *bio*B, *lux*S, *lux*R and many more were present in the two test isolates of *P. stuartii* which were predicted to be responsible for the pathogenicity and had putative role in burn wound infection dynamics.

### Subsystems, metabolic pathways, prophage and CRISPR/Cas system investigation

The subsystems coverage and feature count analysis through RAST server showed that both isolates contained 344 subsystems. The subsystem feature counts and category distribution are not highly different in the two isolates (Supplementary Fig. 2). The metabolic pathway analysis revealed a plethora of pathways having prominent functional impacts. Several pathways were detected having potentiality of different xenobiotic/toxic pollutants biodegradations such as bisphenol A, DDT, toluene, naphthalene, tetrachloroethene and 2, 4-dichlorobenzoate which denoted the probable capacity of the isolates to grow on such harsh environmental conditions indicating toward the potency of the rigidness of the isolates. The isolates also harbor pathways involved in different secondary metabolite biosynthesis such as zeatin, anthocyanin, carotenoid, tropane, flavonoid, biterpenoid, phenylpropanoid and many more. The detailed pathways of both isolates are listed in Supplementary Table 3 and 4. For both isolates, multiple regions of phage integration were identified. *P. stuartii* SHNIBPS63 contained 8 regions of phage integration and *P. stuartii* SHNIBPS71 contained 6 regions (Supplementary Fig. 4). The prophage regions detected belonged to bacteriophage family *Myoviridae* and *Siphoviridae*. On the other hand, in our study, *P. stuartii* SHNIBPS63 was found to harbor class 1 I-F type CRISPR/Cas system in its genome (Supplementary Fig. 3). This CRISPR/Cas system could possibly function toward the resistance against phage as previously described in case of *Enterobacteriaceae*.

### Genome wide antimicrobial resistance gene pool investigation

**Figure 5:**
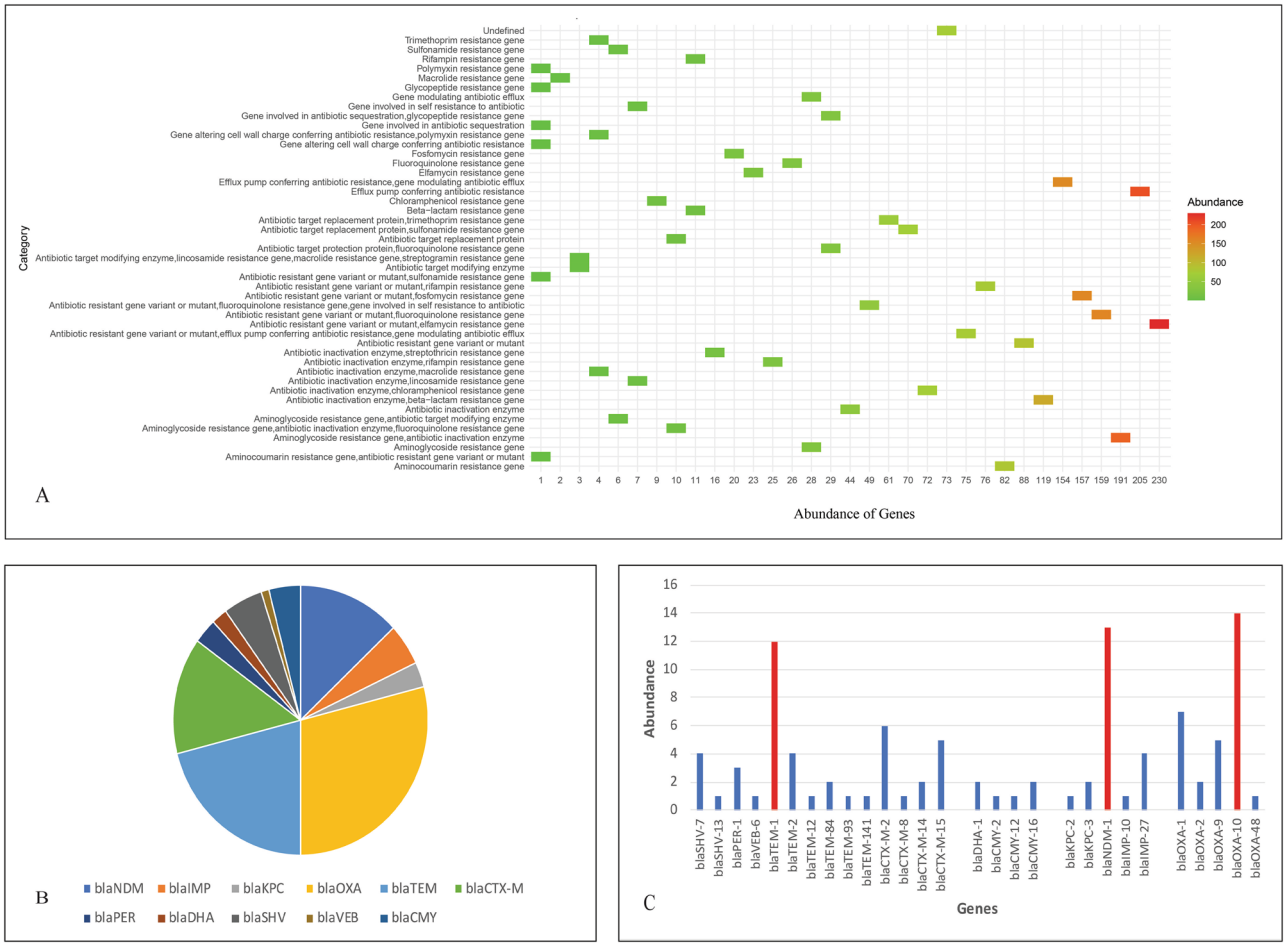
Antimicrobial resistance genes analysis of publicly available 86 genomes of *P. stuartii*. (**A**) Abundance of antimicrobial resistance genes based on categories. (**B**) Abundance of different type of beta lactamase genes in *P. stuartii*. (**C**) Abundance of different variants of beta lactamase genes in *P. stuartii* where the high occurrence of *bla*_OXA-10,_ *bla*_NDM-1_ and *bla*_TEM-1_ is depicted with red bars.

The genome wide analysis of 86 whole genome sequences of *P. stuartii* available in public repositories (NCBI and BV-BRC) including the two isolates of this study, revealed the notable presence of 47 different categories of antimicrobial resistance genes (Fig. 5A). The abundance investigation revealed antibiotic resistance gene variant or mutant (Elfamycin resistance gene), antibiotic inactivation enzyme (Aminoglycoside resistance genes) and efflux pump genes to be the most abundant categories of antimicrobial resistance genes in case of *P. stuartii*. The noteworthy prevalence of diverse categories of antimicrobial resistance genes in *P. stuartii* highlighted a concerning global issue, with a growing pattern of antimicrobial resistance that is becoming increasingly alarming. Further, the abundance analysis of beta lactam resistance genes revealed high abundance of *bla*_OXA_, *bla*_TEM_, *bla*_CTX-M_ and *bla*_NDM_ genes respectively (Fig. 5B). All the beta lactamase genes harbored by the 86 isolates of *P. stuartii* are listed in Supplementary Table 5. An in-depth analysis brought to light the presence of various classes and variants of antimicrobial resistance genes, particularly beta-lactamases (Fig. 5C). Class D beta lactamase gene *bla*_OXA-10_ was found to be most abundant and was followed by *bla*_NDM-1_ and *bla*_TEM-1_. The high occurrence of *bla*_NDM-1_ is highly alarming as this gene can confer resistance to almost all the beta lactam antibiotics including carbapenems. As, *P. stuartii* is intrinsically resistant to many antibiotics including colistin, carbapenems are considered to be the last resort of antibiotics to fight this pathogen. So, high abundance of carbapenem resistance genes like *bla*_NDM-1_ in *P. stuartii* is highly alarming. Genome wide analysis also disclosed the presence of *bla*_NDM-1_ in *P. stuartii* F3W which was isolated from a goose in China. The potential for the transmission of carbapenem-resistant *P. stuartii* with carbapenemase genes between different species poses a substantial threat to public health. This underscores the need for One Health approach to address this issue.

### Pangenome analysis revealed an open pangenome of *Providencia stuartii*

**Figure 6:**
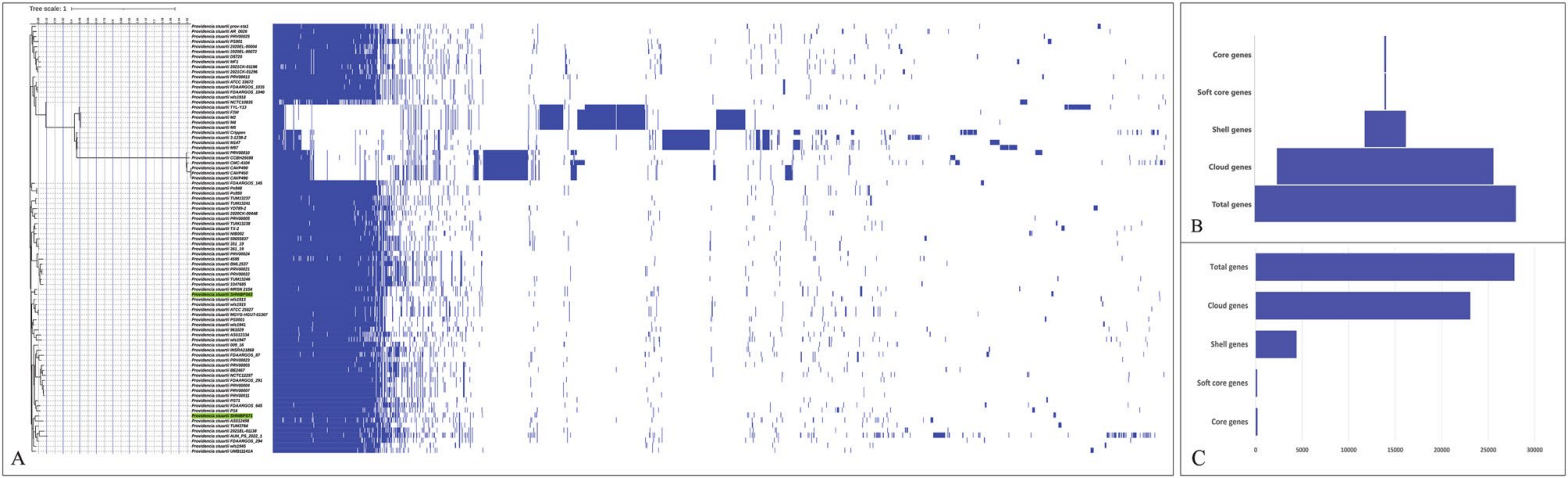
Pangenome and comparative genome analysis. (**A**) Pangenome and pangenome based phylogenetic analysis of 86 different strains of *P. stuartii* worldwide including the two test isolates of this study. (**B**) Comparative scenario of the core and cloud/accessory genome in the pangenome of *P. stuartii*. (**C**) Comparative analysis of each part of the pangenome showing respective number of genes.

**Figure 7:**
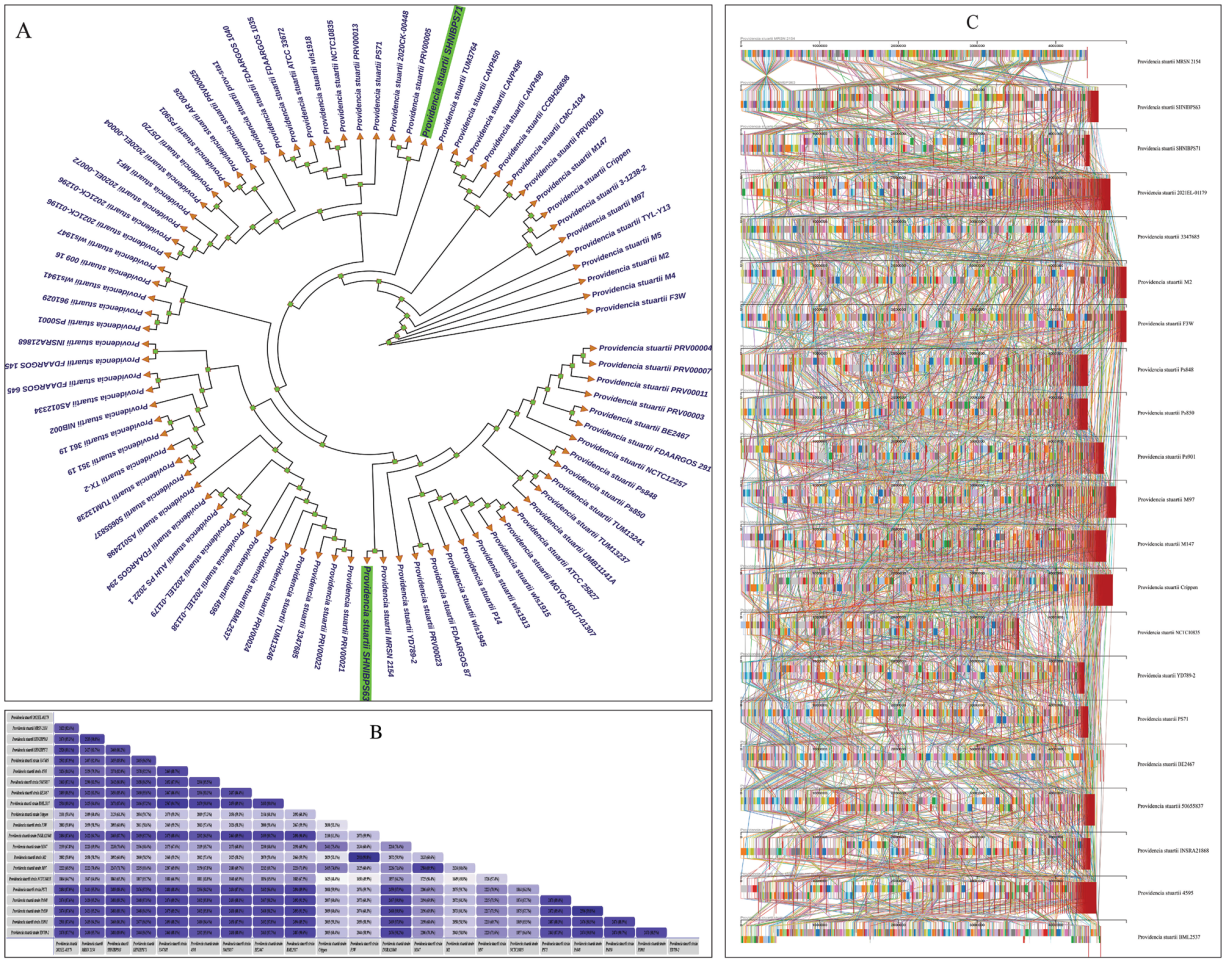
(**A**) SNP-based phylogenomic analysis of all the 86 genomes of *P. stuartii* worldwide including the two test isolates of this study. (**B**) Comparative genomic feature analysis of the representative 21 isolates of *P. stuartii* from different countries and sources including the two test isolates of this study. (**C**) Genome alignment of the genomes using Mauve depicting the variability in the genomes of *P. stuartii*.

For pangenome analysis, total 86 genomes including the two isolates analyzed in this study were selected. The pangenome analysis revealed the core genome being significantly short having only 195 core genes. The too short core genome which is indicative toward the high genomic diversity of *P. stuartii* worldwide (Fig. 6). Total number of genes predicted through pangenome analysis were 27,818 including 165 soft core genes, 4392 shell genes and 23,066 cloud genes (Supplementary Table 6). From pangenome analysis, a too small core genome and large accessory genome clearly depicted that the pangenome of *P. stuartii* is still open which implies ongoing gene acquisition and diversity. An open pangenome arises when a particular taxonomic lineage continues to expand its repertoire of new gene families, and this augmentation does not exhibit asymptotic behavior, irrespective of the number of newly added genomes to the pangenome. The continuous acquisition of genes is a characteristic that could lead to heightened virulence and alterations in the antimicrobial resistance profile of *P. stuartii*. Moreover, the sequencing of additional strains is anticipated to reveal novel genes, potentially giving rise to the adaptation of new mechanisms pertaining to pathogenicity, virulence, and antimicrobial resistance. The pangenome based phylogenetic analysis revealed that the isolates having non-human sources such as *P. stuartii* strain F3W (Isolated from Goose in China), *P. stuartii* strain M2, M4 and M5 (Isolated from Mouse in China), *P. stuartii* TYL-Y13 (Isolated from fermentation dreg in China) formed a clade which were distantly related strains from our two test isolates and most other isolates collected from human source. Another isolate collected from non-human source, *P. stuartii* strain Crippen (Isolated from *Lucilia sericata* in USA, North America) was predicted to be closely related with *P. stuartii* strain 3-1238-2 (Isolated from China) and isolates collected from bathing water in Algeria (*P. stuartii* strain M147 and strain M97). The SNP based phylogenetic analysis also validated the relationship between these strains which are clearly depicted in the rooted tree (Fig. 7A). The SNP based phylogenomic analysis likely provided a more comprehensive representation of the evolutionary lineage and relatedness of the isolates and established a comparative linkage with the genomic characteristics derived from pangenome analysis. From Fig. 7A, it can be clearly observed that *P. stuartii* strain F3W (Isolated from Goose in China) and *P. stuartii* strain M2 (Isolated from Mouse in China) are the isolates which can be denoted as basal taxa from evolutionary aspect. These two isolates shared a common ancestry and through the analysis, they are predicted to be diverged early in the evolutionary history. Following that, the evolution of *P. stuartii* strains M2 and M4 was observed, both of which were isolated from mice in China. Subsequently, another strain, *P. stuartii* strain TYL-Y13, isolated from fermentation dregs in China, was deduced to have undergone evolutionary processes. Despite some ambiguity, based on the assessment of evolutionary relationships through pangenomic and phylogenomic analyses, it can tentatively be inferred that the potential origin of the ancestral lineage of *P. stuartii* may be associated with geographical regions proximate to China. Regarding *P. stuartii* SHNIBPS63, analyses based on both the pangenome and SNP-derived phylogenetic assessments indicated a close relationship between this isolate and *P. stuartii* MRSN2154, substantiating this connection in both genomic and evolutionary contexts. On the other hand, regarding *P. stuartii* SHNIBPS71, a close genomic association was observed with *P. stuartii* AS012498 which was isolated from USA (Fig. 6A). However, in terms of evolutionary relationships, the isolate showed a close relationship with *P. stuartii* PRV00005 and *P. stuartii* 2020CK-00448 which were isolated from USA (Fig. 7A). Phylogenomic analysis also pointed out the divergence of *P. stuartii* of non-human sources with the clinical isolates of *P. stuartii* collected from human sources. From the comparative genomic feature diversity analysis (Fig. 7B, C), it could clearly be observed that the genomes of *P. stuartii* from different continents and countries were significantly diversed from each other having a small (Including the isolates from non-human sources) and moderate (considering only the strains isolated from human sources) number of genes within the core genome.

### Genome wide pathogenicity profiling and proteome comparison

**Figure 8:**
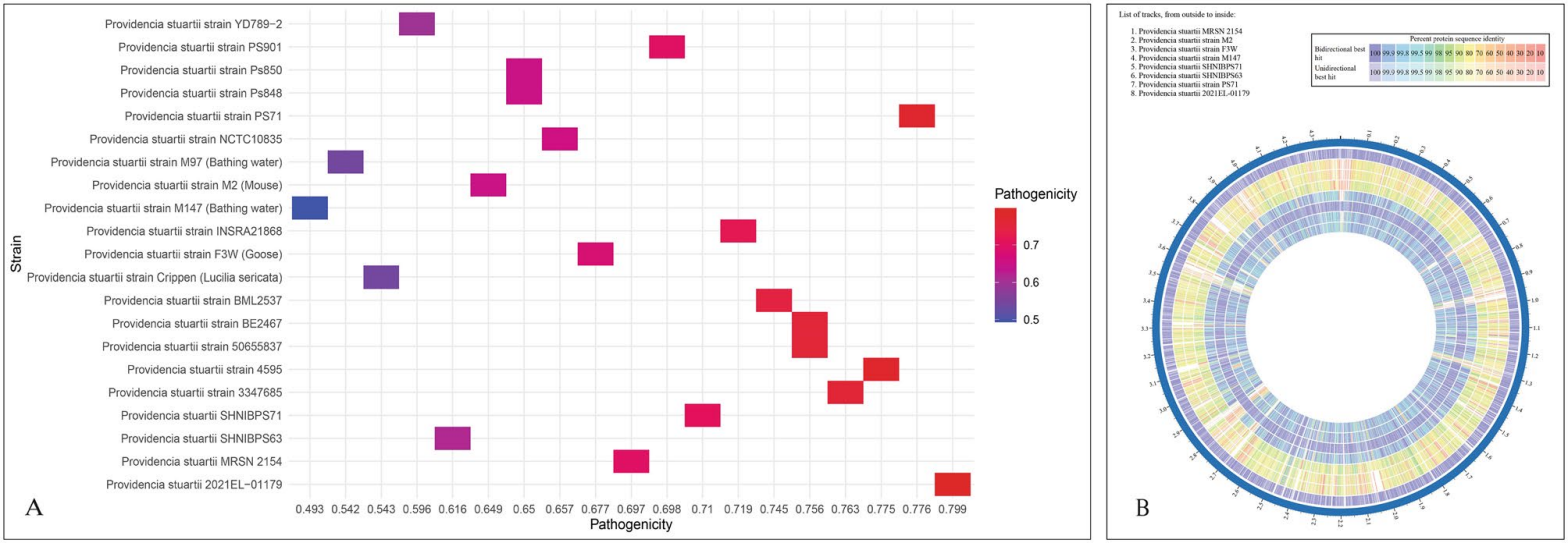
Genome wide pathogenicity analysis of the representative 21 isolates of *P. stuartii* from different countries and sources including the two test isolates of this study. (**A**) Pathogenicity profiling of the isolates based on whole genome sequence analysis. (**B**) Whole proteome comparison of pathogenic and non-pathogenic *P. stuartii* isolated from different sources.

The genome wide pathogenicity profiling of the 21 representative isolates of *P. stuartii* from different sources and countries revealed distinguished pathogenic pattern (Fig. 8). The most pathogenic strain was predicted to be *P. stuartii* strain 2021EL-01179. All the isolates were predicted as human pathogen except three (*P. stuartii* strain Crippen, *P. stuartii* strain M147 and strain M97) which were isolated from non-human sources. These three strains also revealed close genomic and phylogenetic relationship which was discussed in the previous section. This indicated toward the prediction and identification of the non-pathogenic lineage of *P. stuartii*. On the other hand, another two isolates from non-human sources, *P. stuartii* strain F3W (Isolated from Goose in China), *P. stuartii* strain M2 (Isolated from Mouse in China), revealed high pathogenic pattern. The strains of *P. stuartii* isolated from non-human sources showed quite similar proteomic construction when compared with each other and was distinguishable with the strains isolated from human sources. Inspite of being distantly related from the isolates derived from human sources and having a different genomic and proteomic organization, these two isolates were predicted to be human pathogen. As the different strains of *P. stuartii* isolated from both human and non-human sources showed high pathogenic pattern, it can be predicted that this particular organism might be capable of moving between different host species, which could have implications for public health and the understanding of microbial diversity and adaptation across various environments.

## Conclusion

Infections with carbapenem-resistant *Providencia* species greatly affect patient morbidity, mortality and treatment. To our knowledge, this is the first detailed genomic report from Bangladesh about the occurrence of *Providencia stuartii* (*P. stuartii*) infecting burn wounds of burn patients in Bangladesh. The evidence of being resistant to carbapenems indicate toward an alarming condition as carbapenems are considered to be the last line drug to treat *P. stuartii*. The high virulence, pathogenicity, invasive properties, strong biofilm forming capability and multidrug resistance pattern indicates this bacterium to be a highly potential threat for infections in burn and immunocompromised patients. Given the notable high pathogenicity exhibited by distinct strains of *P. stuartii* found in both human and non-human origins, it is reasonable to speculate that this organism possesses the potential to transition between various host species, with potential implications for public health. Further researches and detailed studies are required to investigate the prevalence of *P. stuartii* in Bangladesh as well as emphasize toward unveiling the pathogenicity and resistance mechanism along with finding possibly better and alternative treatment options.

## Material and methods

### Sample collection and identification

The two wound samples were collected from two severely burned patients of 36 years and 45 years old, respectively. Both patients were male and admitted in Sheikh Hasina National Institute of Burn and Plastic Surgery (SHNIBPS), Dhaka, Bangladesh. The wound swabs collected from the patients were immediately processed within one hour and first cultured on MacConkey agar (Oxoid Limited, Basingstoke, UK) and Mueller Hinton agar (Oxoid Limited, Basingstoke, UK) plates in the Microbiological Laboratory. The predominant isolated colonies screened out from MacConkey agar plates were inoculated on nutrient broth (Oxoid Limited, Basingstoke, UK) carefully and brought to the Microbial Genetics and Bioinformatics Laboratory (Lab 109 and 121), Department of Microbiology, University of Dhaka in a cooler with icepacks (below 4 °C). Following isolation, gram staining and a series of biochemical tests were carried out for presumptive identification of the isolates^32^. The extracted chromosomal DNA of the selected isolates was used for the amplification of 16S rDNA by Polymerase Chain Reaction (PCR) using universal primers 27F (5'-AGAGTTTGATCCTGGCTCAG-3') and 1492R (5'-GGTTACCTTGTTACGACTT-3')^33^. The 16S rDNA sequence based identification was carried out using SpeciesFinder2.0^34^ and Nucleotide Blast (BLASTn) tool^35^.

### Antimicrobial susceptibility test and cultural characterization

The two selected isolates of *P. stuartii* were tested for antimicrobial susceptibility following Kirby Bauer method^36^ to 22 antibiotics using the agar disc diffusion method on Mueller–Hinton agar (Oxoid Limited, Basingstoke, UK) following Clinical and Laboratory Standards Institute (CLSI) guidelines (Clinical and Laboratory Standards Institute, 2021)^37^. In case of colistin, MIC (Minimum Inhibitory Concentration) was carried out in order to find out the antimicrobial susceptibility pattern according to the CLSI guidelines. Modified Hodge Test was used to detect the production of carbapenemase in the selected two isolates following standard protocol^38^. Biofilm formation was estimated in 96-well polystyrene microtiter plates (Corning Costar, Waltham, Massachusetts, USA) following crystal violet biofilm formation assay (CV assay)^39^. The antibacterial effect of heavy metals was evaluated in vitro for the isolated pathogens using agar well diffusion method^40^. Three heavy metals such as copper (Cu), zinc (Zn) and iron (Fe) of different concentrations (0.01 M, 0.05 M, and 0.1 M) were used as salts, respectively to study the level of zone of inhibition (ZoI).

### Whole genome sequencing, quality control, assembly and identification

The whole genome sequencing of the two target isolates was performed under Illumina platform using Illumina Miniseq sequencing system at Genome Research Laboratory, Bangladesh Council of Scientific and Industrial Research (BCSIR). The generated FASTQ files were evaluated for quality through FastQC (v0.11)^41^. Further, the adapter sequences and low quality ends per reading were trimmed using Trimmomatic (v0.39)^42^ by setting a criteria having minimum average quality score of 20 and a minimum read length of 50 bp. After trimming, the high quality reads were subjected to de novo assembly using SPAdes v3.15.4 (Species Prediction and Diversity Estimation)^43^. The identification of the isolates were carried out using k-mer algorithm through KmerFinder tool of Center for Genomic Epidemiology^44^. The identification was also carried out and validated based on ANI (Average Nucleotide Identity). ANI Calculator tool (http://enve-omics.ce.gatech.edu/ani/, access date: 2 September, 2023) developed by the Environmental Microbial Genomics Laboratory (enve-omics lab) at the Georgia Institute of Technology was used for calculating the average nucleotide identity of our test isolates with two reference isolates of *Providencia stuartii*; *Providencia stuartii* MRSN 2154 (Accession no. GCF_000259175.1) and *Providencia stuartii* ATCC 33672 (Accession no. GCF_000754345.1)^45^.

### Genome annotation, pathogenicity detection and genomic organization mapping

Due to the infrequent reporting of whole genome sequences of *P. stuartii* and the limited number of available genomes in public databases, relying on a singular algorithm or pipeline for annotation may not comprehensively cover and assign functions to all potential coding sequences (CDS) or genes. So, the assembled sequences for the two isolates were undergone annotation through multiple algorithms or pipelines such as Prokka^46^, RAST^47^, eggNOG^48^ and Prokaryotic genome annotation pipeline (PGAP) of NCBI. Prokka depends on external feature prediction tools such as Prodigal, RNAmmer, Aragorn, SignalP and Infernal to predict and identify the coordinates of genomic features within contigs. On the other hand, eggNOG is focused on functional annotation and metabolic pathways analysis where orthologous groups are functionally annotated through updated versions of Gene Ontology. PGAP performs structural annotation by aligning open reading frames (ORFs) with libraries of protein hidden Markov models (HMMs), representative RefSeq proteins, and proteins sourced from thoroughly characterized reference genomes. For genomic regions lacking HMM or protein evidence, GeneMarkS-2+ generates ab initio predictions of coding regions and determines start sites for ORFs supported by HMM evidence. Prediction of the two bacterial isolate’s pathogenicity towards human hosts was carried out through PathogenFinder (v1.1)^49^ webtool of Center for Genomic Epidemiology. Additionally, the sources of each contigs were investigated through SourceFinder^50^ tool of the same platform. The graphical maps of the circular genomes were generated through Proksee server^51^, which is an expert system for genome visualization.

### Investigation of antimicrobial resistance genes, virulence factors gene and metabolic functions profiling

Antimicrobial resistance genes were investigated through CARD^52^ and ResFinder^53^. The virulence factor genes were explored with Victors^54^, which is a novel, manually curated, web-based integrative knowledge base and analysis resource for VFs of pathogens that cause infectious diseases in human and animals. The metabolic functional potentials of the genomes were predicted through the Patric server pathway analysis^55^. The metal resistance genes were analyzed through BacMet^56^, an easy-to-use bioinformatics resource of antibacterial biocide- and metal-resistance genes.

### Mobile genetic elements (MGEs), prophage and CRISPR/Cas system investigation

The MGEs from the whole genomes of the isolates were investigated through MGEFinder^57^ and mobileOG-db^58^. Following the MGEs investigation, integrons were searched from the sequences using IntegronFinder^59^ tool. De novo plasmid assembly from the whole genome sequence was carried out through plasmidSpades^60^. Phage integration inside the bacterial genomes as well as in the plasmids were inspected through PHAge Search Tool Enhanced Release (PHASTER)^61^ webserver and Phigaro^62^ tool. CRISPR/Cas systems in the bacterial whole genomes were investigated through CRISPR/Cas Finder algorithm^63^.

### Genome wide antimicrobial resistance gene and pangenome analysis

Genome wide antimicrobial resistance gene pool analysis included all the 86 (n = 86) whole genomes of *P. stuartii* available in NCBI and BV-BRC database including the two isolates of this study. The whole genome sequences were downloaded in FASTA format and CARD Resistance Gene Identifier^52^ was used to analyze the antimicrobial resistance genes from the genomic data. Following that, all the 86 genomes were considered for pangenome analysis in order to find out the diversity of the target strains and aimed to reveal gene or gene family presence absence variations (PAVs) among strains from different origin and country. Annotation of the genomes were carried out using Prokka^46^. The gff3 files were subjected for pangenome analysis using Roary^64^, a tool that rapidly builds large scale pan genomes identifying the core and accessory genes. Microbial entities have the capacity to swiftly assimilate genes from diverse organisms, potentially augmenting virulence or fostering resistance to antimicrobial drugs. Enhancing comprehension of an organism's conserved genes and its accessory genome provides valuable insights into fundamental processes like selection and evolution. As the detailed genomic feature of *P. stuartii* is being newly documented in Bangladesh through this investigation, we employed Roary to obtain a comprehensive overview of the organism's pangenome status as well as to find out the genomic diversity of the Bangladeshi strains with all other strains reported worldwide.

### Whole genome-based phylogenetic analysis and pathogenicity profiling

A comprehensive investigation of the phylogenomic relationships among the total 86 (n = 86) publicly available *P. stuartii* genomes, inclusive our two test isolates, was conducted through an analysis of whole genome sequences which incorporated multiple reference sequences along with a single nucleotide polymorphism (SNP)-based approach. The genome sequences were downloaded in both fasta and fastq format and then processed for phylogenetic analysis. REALPHY^65^, a pipeline that can infer phylogenetic trees from whole genome sequence data were used to conduct the phylogenomic analysis in this study. In this pipeline, all provided sequences (references and queries) are mapped to each of the references via bowtie2^66^. After that, from the multiple sequence alignment data along with SNPs and deleted sites, phylogenetic tree is constructed and inferred via PhyML. After conducting the phylogenomic analysis through REALPHY, a rooted phylogenetic tree was created with iTOL^67^ in order to comprehend the evolutionary lineage and relatedness of the isolates.

Among all the 86 whole genome sequences, representative 21 (n = 21) genomes from different continents such as Europe (n = 5), South America (n = 4), North America (n = 4), Africa (n = 2), Asia (n = 5) and sources (Human = 14, Non-human = 6, Reference = 1) including the two isolates of this study were selected for comparative pathogenicity profiling. Pathogenicity profiling and prediction of the pathogenicity of all the isolates towards human hosts was carried out through PathogenFinder (v1.1)^49^ webtool of Center for Genomic Epidemiology and analyzed as well as visualized using Tidyverse package of RStudio^68^. PathogenFinder is a pipeline for the prediction of bacterial pathogenicity by analyzing the input proteome, genome, or raw reads. The approach is not biased on sets of genes known to be associated with pathogenicity, rather the approach could aid the discovery of novel pathogenicity factors as well. The method includes a comparison process assessing the input proteins against the PFDB (Protein family database), followed by the application of an identity threshold to filter the hits. Subsequently, a final score is computed by summing the Z values associated with the identified PFs. This final score is then juxtaposed against the model's ZTHR threshold to arrive at the ultimate prediction^69^. Further, the genomes were aligned and visualized using Mauve software package which does alignment of conserved genomic sequence with rearrangements^70^. Also, the entire set of annotated genes of each genome were compared in the basis of presence and absence through Funrich^71^ software. Following the pathogenicity analysis, eight representative genomes were selected from both pathogenic and non-pathogenic category to compare their whole proteome through bidirectional BLASTP in order to comprehend and correlate with their pathogenicity profile.

### Ethical permission

Ethical permission to conduct the study was given and verified by the Ethical Review Committee of Faculty of Biological Sciences, University of Dhaka (Ref. No. 191/Biol. Scs.). We confirm that all methods were performed in accordance with the relevant guidelines and regulations. Informed consent was taken from the patients for sample collection, conduct research with the samples and to publish the outcome.

### Supplementary Information

Supplementary Information.

## Data Availability

The draft genome sequences of the two isolate *Providencia stuartii* SHNIBPS63 and *Providencia stuartii* SHNIBPS71 were deposited in DDBJ/ENA/GenBank of NCBI under the accession number JASDEX000000000 (https://www.ncbi.nlm.nih.gov/nuccore/JASDEX000000000.1/) and JASDEW000000000 (https://www.ncbi.nlm.nih.gov/nuccore/JASDEW000000000.1/) respectively. The data were made publicly available. Besides, the other genomes used for secondary analysis were retrieved from NCBI (https://www.ncbi.nlm.nih.gov/) and BV-BRC (https://www.bv-brc.org/).
