# Supplementary material for: Genomic landscape of NDM-1 producing multidrug-resistant Providencia stuartii causing burn wound infections in Bangladesh
Source: Sci Rep. 2024 Jan 26;14:2246. doi: 10.1038/s41598-024-51819-9 (PMC10817959; doi:10.1038/s41598-024-51819-9)
Supplement: Supplementary file 1 — Supplementary Information. [file 41598_2024_51819_MOESM1_ESM.pdf]

Supplementary Information File

Genomic landscape of NDM-1 producing multidrug-resistant *Providencia stuartii* causing burn wound infections in Bangladesh

Spencer Mark Mondol[1], Israt Islam[1], Md. Rafiul Islam[1], Shahriar Kabir Shakil[1,5], Nadira Naznin Rakhi[1], Jannatul Ferdous Mustary[2], Amiruzzaman[3], Donald James Gomes[1], Hussain Md. Shahjalal[4], Md. Mizanur Rahaman[1*]

[1]Department of Microbiology, University of Dhaka, Dhaka-1000, Bangladesh

[2]Microbiology Department, Sheikh Hasina National Institute of Burn and Plastic Surgery, Dhaka-1000, Bangladesh

[3]Department of Medicine, Sir Salimullah Medical College, Dhaka-1000, Bangladesh

[4]Department of Biochemistry and Molecular Biology, Jahangirnagar University, Savar, Dhaka-1342, Bangladesh

[5]Department of Biotechnology and Genetic Engineering, Noakhali Science and Technology University, Noakhali-3814, Bangladesh

*Corresponding Author

Corresponding to:

Md. Mizanur Rahaman, PhD

Associate Professor

Department of Microbiology,

University of Dhaka, Dhaka-1000, Bangladesh

Email: razu002@du.ac.bd

Supplementary Fig 1: Antibiotic susceptibility pattern of the two test isolates. (PS63= *Providencia stuartii* SHNIBPS63 and PS71= *Providencia stuartii* SHNIBPS71)

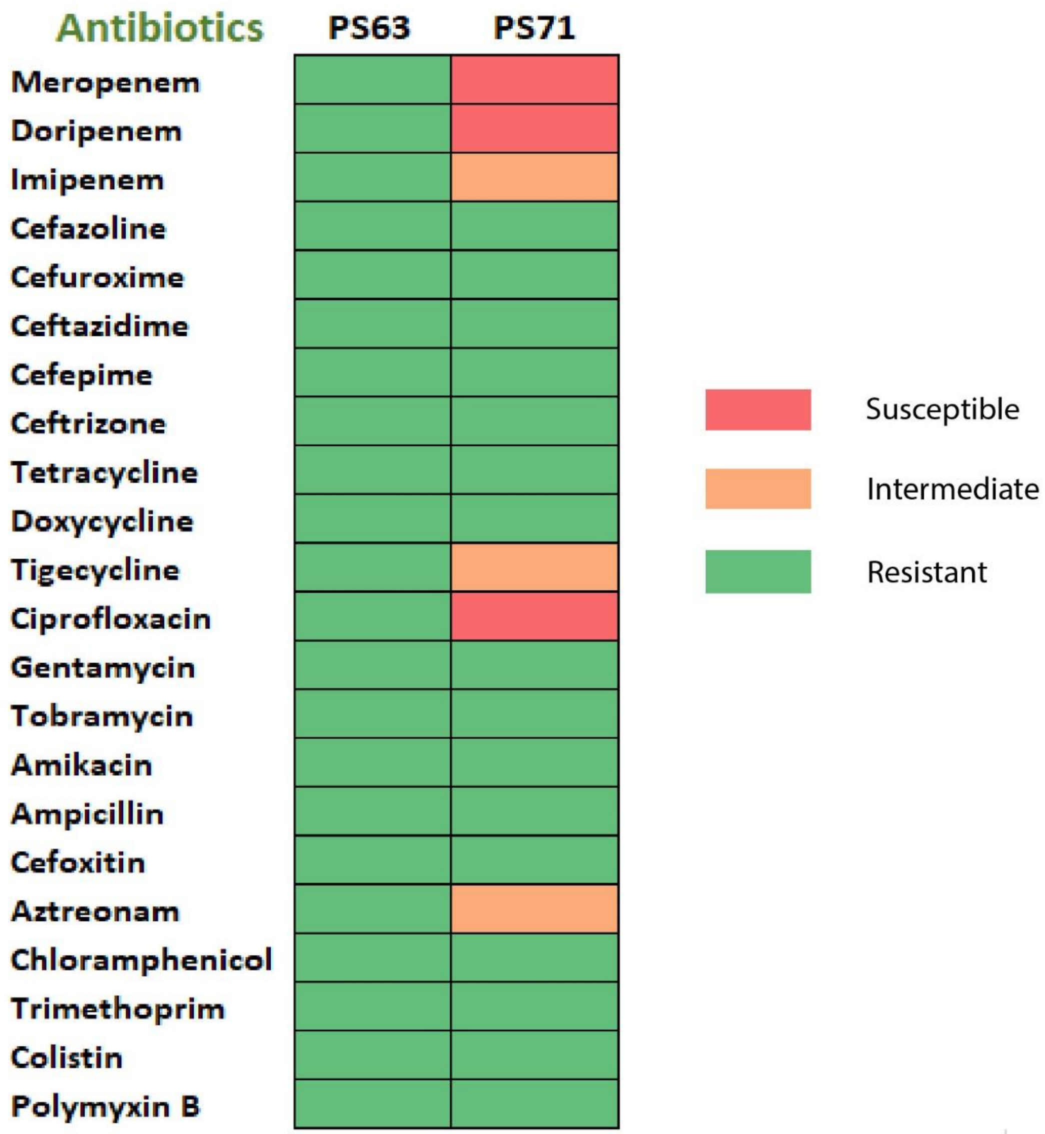

Supplementary Fig 2: Subsystem prediction and analysis through RAST server revealing subsystem coverage, distribution and feature counts in *P. stuartii* SHNIBPS63 (A) and *P. stuartii* SHNIBPS71 (B)

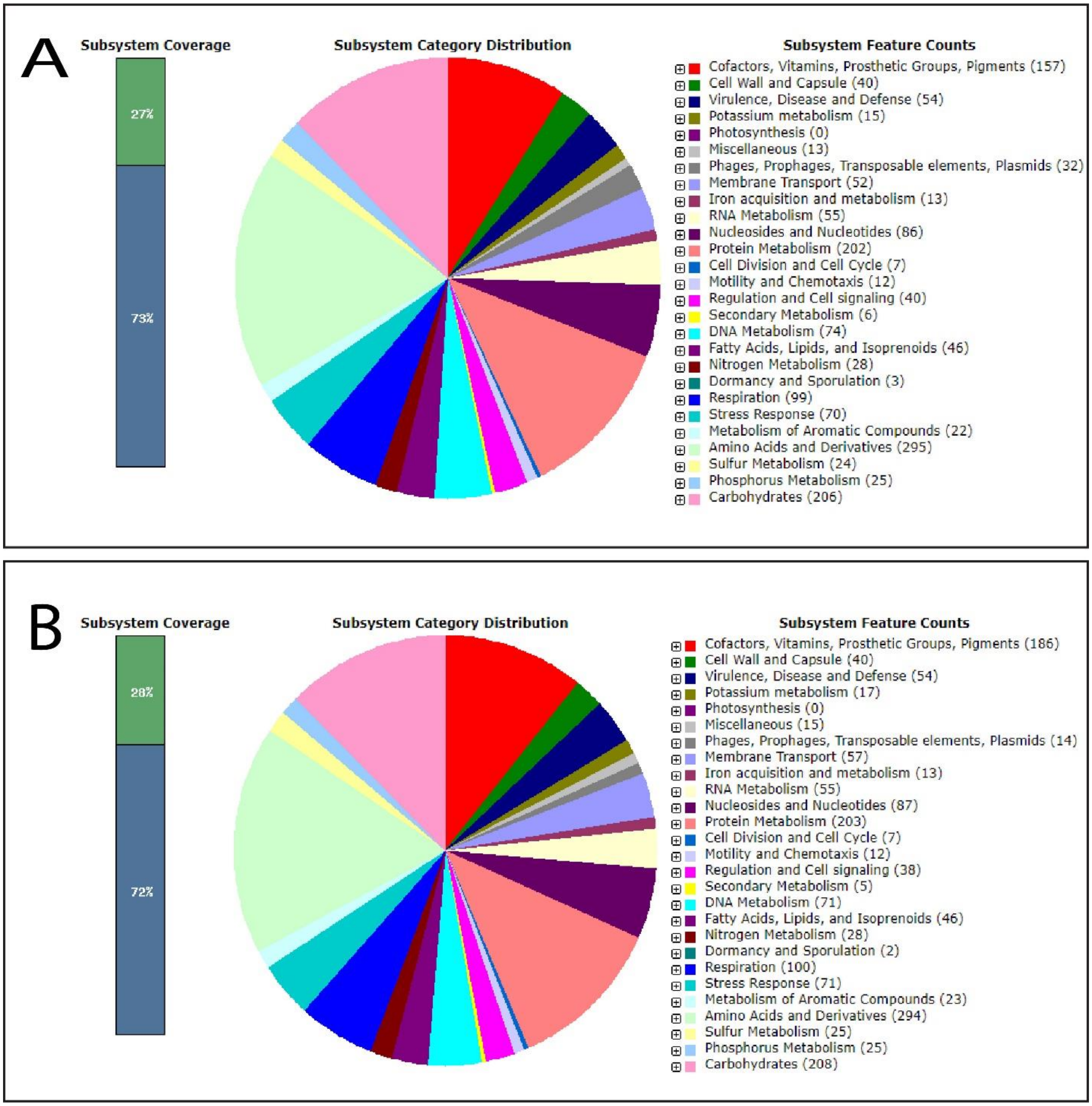

Supplementary Fig 3: The organization and mapping of class 1 I-F type CRISPR/Cas system in the genome of *P. stuartii* SHNIBPS63

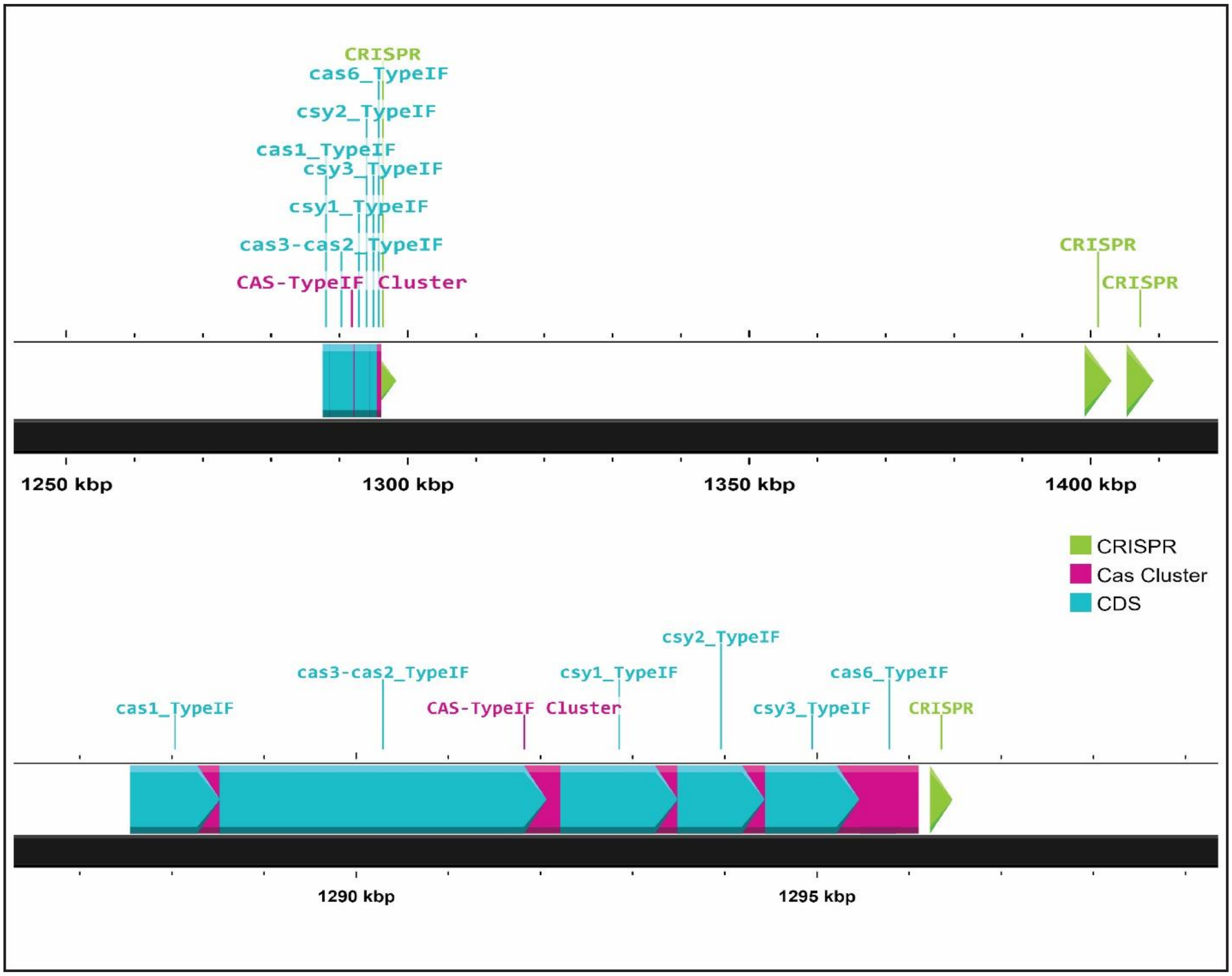

Supplementary Fig 4: The prophages/phage sequences integrated into the bacterial genomes of *Providencia stuartii* SHNIBPS63 (A,B) and *Providencia stuartii* SHNIBPS71 (C,D)

A

Region	Region Length	Completeness	Score	# Total Proteins	Region Position	Most Common Phage	GC %
NODE_1_length_839059_cov_14.885247							
1	16.5Kb	questionable	70	22	223093-239642	PHAGE_Haemop_Aaphi23_NC_004827(10)	40.19%
2	16.2Kb	incomplete	40	19	739942-756145	PHAGE_Bacill_G_NC_023719(2)	39.63%
NODE_2_length_390297_cov_21.352327							
3	32.9Kb	intact	100	27	238304-271249	PHAGE_Staphy_SPbeta_like_NC_029119(1)	41.28%
NODE_3_length_383033_cov_15.262492							
4	40.3Kb	questionable	70	42	120559-160943	PHAGE_Pectob_ZF40_NC_019522(13)	30.45%
NODE_5_length_293933_cov_17.814586							
5	31.8Kb	incomplete	40	19	248836-280668	PHAGE_Escher_500465_1_NC_049342(7)	38.77%
NODE_10_length_162460_cov_18.126331							
6	22Kb	incomplete	60	23	117149-139214	PHAGE_Entero_UAB_Phi20_NC_031019(2)	40.27%
7	27.7Kb	questionable	70	59	131090-158834	PHAGE_Pseudo_phiPSA1_NC_024365(6)	41.72%
NODE_12_length_146884_cov_17.213110							
8	37.4Kb	incomplete	40	26	59991-97402	PHAGE_Entero_P4_NC_001609(3)	40.94%

B

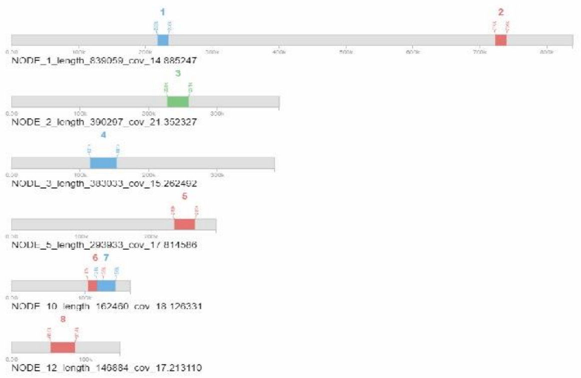

C

Region	Region Length	Completeness	Score	# Total Proteins	Region Position	Most Common Phage	GC %
NODE_1_length_807101_cov_9.572121							
1	59.9Kb	intact	150	69	254700-314654	PHAGE_Salmon_Fels_2_NC_010463(12)	40.00%
2	32.8Kb	incomplete	40	29	546387-579230	PHAGE_Entero_P4_NC_001609(3)	40.96%
NODE_2_length_678265_cov_9.727163							
3	23.8Kb	incomplete	30	16	211746-235644	PHAGE_Escher_500465_1_NC_049342(7)	39.31%
NODE_3_length_520156_cov_8.661980							
4	19.7Kb	questionable	70	30	483192-502932	PHAGE_Haemop_Aaphi23_NC_004827(10)	38.70%
NODE_13_length_103981_cov_15.641871							
5	13.2Kb	incomplete	20	10	77439-90716	PHAGE_Salmon_SJ46_NC_031129(2)	54.07%
NODE_15_length_90757_cov_10.926202							
6	43.1Kb	intact	150	77	214-43403	PHAGE_Entero_ES18_NC_006949(13)	42.38%

D

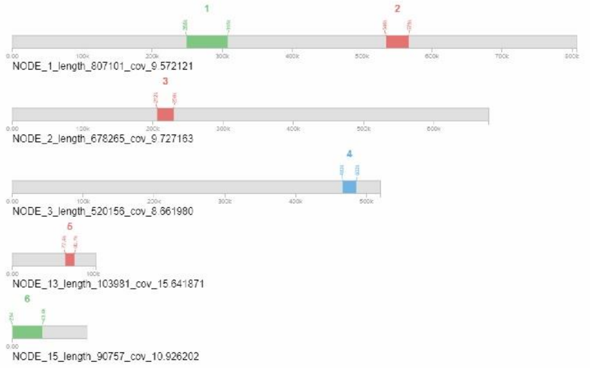

Supplementary Table 1: List of the metal and biocide resistance genes present in *P. stuartii* SHNIBPS63 and *P. stuartii* SHNIBPS71

P. stuartii SHNIBPS63	*P. stuartii* SHNIBPS71
acrA	acrA
acrB	acrB
envC	envC
acrR	acrF
actP	acrR
arsA	actP
arsB	arsA
arsC	arsB
arsD	arsC
arsR	arsD
baeR	arsR
baeS	baeR
bcr	baeS
copA	bcr
corC	copA
cpxA	corC
cpxR	cpxA
cueO	cpxR
cueR	cueO
cutA	cueR
cutC	cutA
cutF	cutC
dps	cutF
dsbA	dps
dsbB	dsbA
dsbC	dsbB
emrA	dsbC
emrB	emrA
emrD	emrB
emrE	emrD
fabI	emrE
fbpC	fabI
ybbL	fbpC
ybbM	ybbL
fieF	ybbM
fptA	fieF
galE	fptA

glpF	galE
hdeA	glpF
hdeB	hdeA
ibpA	hdeB
iclR	ibpA
mdeA	iclR
mdtA	mdeA
mdtB	mdtA
mdtC	mdtB
mdtG	mdtC
mdtI	mdtG
mdtJ	mdtI
mdtK	mdtJ
merA	mdtK
merD	mntP
merE	modA
merP	modB
merT	modC
mntP	modE
modA	norM
modB	lptD
modC	pcm
modE	phoB
norM	phoR
lptD	pitA
pcm	pstA
phoB	pstB
phoR	pstC
pitA	pstS
pstA	yohM
pstB	recG
pstC	rpoS
pstS	ruvB
yohM	sitA
recG	sitB
rpoS	sitC
ruvB	smvA
sitA	sodA
sitB	sodB
sitC	soxR
smvA	soxS

sodA	sugE
sodB	tehB
soxR	terA
soxS	terB
sugE	terC
tehB	terD
terA	terE
terB	terW
terC	terZ
terD	tolC
terE	tupA
terW	ychH
terZ	ydeP
tolC	yfeD
ychH	ygiW
ydeP	zinT
yfeD	zntA
ygiW	zntR
zinT	znuA
zntA	znuB
zntR	znuC
znuA	zur
znuB	
znuC	
zur	

Supplementary Table 2: List of major fimbrial clusters and chaperone usher fimbriae associated proteins

Fimbriae Type	Associated Proteins
Type I Fimbriae	FimA- Type-I fimbrial protein<br>FimC- Chaperone protein<br>FimD- Outer membrane usher protein<br>FimH- Type 1 fimbrin D-mannose specific adhesin<br>FimG- Fimbrial protein<br>FimF- Fimbrial protein
P Fimbriae	PapA- Fimbrial major pilin protein<br>PapH- Fimbrial minor pilin protein<br>PapK- Fimbrial adapter PapK<br>PapC- Outer membrane usher protein PapC<br>PapD- Chaperone protein PapD
Yfc	YfcA- Putative membrane transporter protein<br>YfcD- Putative nudix hydrolase<br>YfcQ- Putative fimbrial-like protein<br>YfcR- Putative fimbrial-like protein<br>YfcS- Putative fimbrial chaperone<br>YfcP- Putative fimbrial-like protein<br>YfcJ- Putative MFS type transporter
LPF	LpfA- Putative major fimbrial subunit<br>LpfB- Putative fimbrial chaperone
Yad	YadV- Putative fimbrial chaperone<br>YadM- Fimbrial protein<br>YadA- YadA like membrane anchor domain<br>HtrE- Outer membrane usher protein
Yeh	YehB- outer membrane usher protein<br>YehC- putative fimbrial chaperone
Yqi	YgiL- Fimbrial protein<br>YqiG- Usher protein<br>YqiL- Fimbrial protein
Ybg	YbgD- Putative fimbrial like protein<br>YbgP- Fimbrial protein
Yra	YraI- Putative fimbrial chaperone<br>YraJ- Outer membrane usher protein
Others	MrkD- Fimbria adhesin protein<br>SmfA- Fimbria A protein<br>PmfA- Major fimbrial subunit<br>Smf-1- Major fimbrial subunit<br>SfmD- Outer membrane usher protein<br>MrpA- Major MR/P fimbria protein<br>ElfD- Putative fimbrial chaperone protein

Supplementary Table 3: Metabolic pathways of *P. stuartii* SHNIBPS63

**Pathway Name**	**Pathway Class**
Methane metabolism	Energy Metabolism
Glutathione metabolism	Metabolism of Other Amino Acids
Purine metabolism	Nucleotide Metabolism
Porphyrin and chlorophyll metabolism	Metabolism of Cofactors and Vitamins
Sulfur metabolism	Energy Metabolism
Benzoate degradation via hydroxylation	Xenobiotics Biodegradation and Metabolism
Fatty acid metabolism	Lipid Metabolism
Vitamin B6 metabolism	Metabolism of Cofactors and Vitamins
Reductive carboxylate cycle (CO2 fixation)	Energy Metabolism
Fructose and mannose metabolism	Carbohydrate Metabolism
Nitrogen metabolism	Energy Metabolism
Oxidative phosphorylation	Energy Metabolism
Ubiquinone and other terpenoid-quinone biosynthesis	Metabolism of Cofactors and Vitamins
Fatty acid biosynthesis	Lipid Metabolism
Pyrimidine metabolism	Nucleotide Metabolism
Phenylalanine, tyrosine and tryptophan biosynthesis	Amino Acid Metabolism
Novobiocin biosynthesis	Biosynthesis of Secondary Metabolites
Lysine biosynthesis	Amino Acid Metabolism
Drug metabolism - other enzymes	Xenobiotics Biodegradation and Metabolism
Lysine degradation	Amino Acid Metabolism
Selenoamino acid metabolism	Metabolism of Other Amino Acids
Pentose and glucuronate interconversions	Carbohydrate Metabolism
Tetrachloroethene degradation	Xenobiotics Biodegradation and Metabolism
Drug metabolism - cytochrome P450	Xenobiotics Biodegradation and Metabolism
Tyrosine metabolism	Amino Acid Metabolism
Naphthalene and anthracene degradation	Xenobiotics Biodegradation and Metabolism
Glyoxylate and dicarboxylate metabolism	Carbohydrate Metabolism
Citrate cycle (TCA cycle)	Carbohydrate Metabolism
Amino sugar and nucleotide sugar metabolism	Carbohydrate Metabolism
Metabolism of xenobiotics by cytochrome P450	Xenobiotics Biodegradation and Metabolism
Alanine, aspartate and glutamate metabolism	Amino Acid Metabolism
Carbon fixation in photosynthetic organisms	Energy Metabolism
Cysteine and methionine metabolism	Amino Acid Metabolism
Pyruvate metabolism	Carbohydrate Metabolism
Phenylalanine metabolism	Amino Acid Metabolism
Glycerophospholipid metabolism	Lipid Metabolism

Glycerolipid metabolism	Lipid Metabolism
Pentose phosphate pathway	Carbohydrate Metabolism
Aminoacyl-tRNA biosynthesis	Translation
Phosphonate and phosphinate metabolism	Metabolism of Other Amino Acids
Valine, leucine and isoleucine degradation	Amino Acid Metabolism
beta-Alanine metabolism	Metabolism of Other Amino Acids
Phenylpropanoid biosynthesis	Biosynthesis of Secondary Metabolites
alpha-Linolenic acid metabolism	Lipid Metabolism
Histidine metabolism	Amino Acid Metabolism
Tryptophan metabolism	Amino Acid Metabolism
Terpenoid backbone biosynthesis	Biosynthesis of Secondary Metabolites
Cyanoamino acid metabolism	Metabolism of Other Amino Acids
Riboflavin metabolism	Metabolism of Cofactors and Vitamins
1,4-Dichlorobenzene degradation	Xenobiotics Biodegradation and Metabolism
Glycine, serine and threonine metabolism	Amino Acid Metabolism
Propanoate metabolism	Carbohydrate Metabolism
Valine, leucine and isoleucine biosynthesis	Amino Acid Metabolism
Butanoate metabolism	Carbohydrate Metabolism
D-Glutamine and D-glutamate metabolism	Metabolism of Other Amino Acids
One carbon pool by folate	Metabolism of Cofactors and Vitamins
1- and 2-Methylnaphthalene degradation	Xenobiotics Biodegradation and Metabolism
High-mannose type N-glycan biosynthesis	Glycan Biosynthesis and Metabolism
Limonene and pinene degradation	Biosynthesis of Secondary Metabolites
Fatty acid elongation in mitochondria	Lipid Metabolism
Pantothenate and CoA biosynthesis	Metabolism of Cofactors and Vitamins
C5-Branched dibasic acid metabolism	Carbohydrate Metabolism
Tetracycline biosynthesis	Biosynthesis of Secondary Metabolites
Anthocyanin biosynthesis	Biosynthesis of Secondary Metabolites
Ether lipid metabolism	Lipid Metabolism
Isoquinoline alkaloid biosynthesis	Biosynthesis of Secondary Metabolites
Sphingolipid metabolism	Lipid Metabolism
Starch and sucrose metabolism	Carbohydrate Metabolism
Caprolactam degradation	Xenobiotics Biodegradation and Metabolism
Geraniol degradation	Xenobiotics Biodegradation and Metabolism
Arginine and proline metabolism	Amino Acid Metabolism
Glycolysis / Gluconeogenesis	Carbohydrate Metabolism
Galactose metabolism	Carbohydrate Metabolism
Peptidoglycan biosynthesis	Glycan Biosynthesis and Metabolism
Glycosphingolipid biosynthesis - globo series	Glycan Biosynthesis and Metabolism
Glycosphingolipid biosynthesis - ganglio series	Glycan Biosynthesis and Metabolism
Streptomycin biosynthesis	Biosynthesis of Secondary Metabolites

Biotin metabolism	Metabolism of Cofactors and Vitamins
Nicotinate and nicotinamide metabolism	Metabolism of Cofactors and Vitamins
Diterpenoid biosynthesis	Biosynthesis of Secondary Metabolites
Betalain biosynthesis	Biosynthesis of Secondary Metabolites
Lipopolysaccharide biosynthesis	Glycan Biosynthesis and Metabolism
Folate biosynthesis	Metabolism of Cofactors and Vitamins
Ascorbate and aldarate metabolism	Carbohydrate Metabolism
Flavonoid biosynthesis	Biosynthesis of Secondary Metabolites
Stilbenoid, diarylheptanoid and gingerol biosynthesis	Biosynthesis of Secondary Metabolites
Biosynthesis of type II polyketide products	Biosynthesis of Polyketides and Nonribosomal Peptides
Thiamine metabolism	Metabolism of Cofactors and Vitamins
Penicillin and cephalosporin biosynthesis	Biosynthesis of Secondary Metabolites
Phosphatidylinositol signaling system	Signal Transduction
Taurine and hypotaurine metabolism	Metabolism of Other Amino Acids
Biosynthesis of siderophore group nonribosomal peptides	Biosynthesis of Polyketides and Nonribosomal Peptides
Atrazine degradation	Xenobiotics Biodegradation and Metabolism
D-Arginine and D-ornithine metabolism	Metabolism of Other Amino Acids
D-Alanine metabolism	Metabolism of Other Amino Acids
Toluene and xylene degradation	Xenobiotics Biodegradation and Metabolism
Biphenyl degradation	Xenobiotics Biodegradation and Metabolism
Retinol metabolism	Metabolism of Cofactors and Vitamins
Insect hormone biosynthesis	Biosynthesis of Secondary Metabolites
Inositol phosphate metabolism	Carbohydrate Metabolism
Ethylbenzene degradation	Xenobiotics Biodegradation and Metabolism
2,4-Dichlorobenzoate degradation	Xenobiotics Biodegradation and Metabolism
Biosynthesis of type II polyketide backbone	Biosynthesis of Polyketides and Nonribosomal Peptides
Glycosaminoglycan degradation	Glycan Biosynthesis and Metabolism
Styrene degradation	Xenobiotics Biodegradation and Metabolism
Lipoic acid metabolism	Metabolism of Cofactors and Vitamins
Photosynthesis	Energy Metabolism
Arachidonic acid metabolism	Lipid Metabolism
Glycosphingolipid biosynthesis - lacto and neolacto series	Glycan Biosynthesis and Metabolism
Glycosylphosphatidylinositol (GPI)-anchor biosynthesis	Glycan Biosynthesis and Metabolism
Biosynthesis of ansamycins	Biosynthesis of Polyketides and Nonribosomal Peptides
Polyketide sugar unit biosynthesis	Biosynthesis of Polyketides and Nonribosomal Peptides

beta-Lactam resistance	Biosynthesis of Secondary Metabolites
Tropane, piperidine and pyridine alkaloid biosynthesis	Biosynthesis of Secondary Metabolites
1,1,1-Trichloro-2,2-bis(4-chlorophenyl)ethane (DDT) degradation	Xenobiotics Biodegradation and Metabolism
Biosynthesis of 12-, 14- and 16-membered macrolides	Biosynthesis of Polyketides and Nonribosomal Peptides
Primary bile acid biosynthesis	Lipid Metabolism
Carotenoid biosynthesis	Biosynthesis of Secondary Metabolites
Linoleic acid metabolism	Lipid Metabolism
O-Glycan biosynthesis	Glycan Biosynthesis and Metabolism
Zeatin biosynthesis	Biosynthesis of Secondary Metabolites
Flavone and flavonol biosynthesis	Biosynthesis of Secondary Metabolites
Biosynthesis of unsaturated fatty acids	Lipid Metabolism
Bisphenol A degradation	Xenobiotics Biodegradation and Metabolism
Biosynthesis of vancomycin group antibiotics	Biosynthesis of Polyketides and Nonribosomal Peptides
C21-Steroid hormone metabolism	Lipid Metabolism
T cell receptor signaling pathway	Immune System
Secondary bile acid biosynthesis	Lipid Metabolism

Supplementary Table 4: Metabolic pathways of *P. stuartii* SHNIBPS71

**Pathway Name**	**Pathway Class**
Pentose and glucuronate interconversions	Carbohydrate Metabolism
Lysine degradation	Amino Acid Metabolism
Glycolysis / Gluconeogenesis	Carbohydrate Metabolism
Phenylpropanoid biosynthesis	Biosynthesis of Secondary Metabolites
Tryptophan metabolism	Amino Acid Metabolism
1,1,1-Trichloro-2,2-bis(4-chlorophenyl)ethane (DDT) degradation	Xenobiotics Biodegradation and Metabolism
Toluene and xylene degradation	Xenobiotics Biodegradation and Metabolism
Purine metabolism	Nucleotide Metabolism
Tyrosine metabolism	Amino Acid Metabolism
Vitamin B6 metabolism	Metabolism of Cofactors and Vitamins
Aminoacyl-tRNA biosynthesis	Translation
Thiamine metabolism	Metabolism of Cofactors and Vitamins
beta-Alanine metabolism	Metabolism of Other Amino Acids
Cysteine and methionine metabolism	Amino Acid Metabolism
Fructose and mannose metabolism	Carbohydrate Metabolism
Metabolism of xenobiotics by cytochrome P450	Xenobiotics Biodegradation and Metabolism
Drug metabolism - cytochrome P450	Xenobiotics Biodegradation and Metabolism
1,4-Dichlorobenzene degradation	Xenobiotics Biodegradation and Metabolism
Pyruvate metabolism	Carbohydrate Metabolism
Glutathione metabolism	Metabolism of Other Amino Acids
Carbon fixation in photosynthetic organisms	Energy Metabolism
Tropane, piperidine and pyridine alkaloid biosynthesis	Biosynthesis of Secondary Metabolites
Arginine and proline metabolism	Amino Acid Metabolism
Phenylalanine metabolism	Amino Acid Metabolism
Insect hormone biosynthesis	Biosynthesis of Secondary Metabolites
Phenylalanine, tyrosine and tryptophan biosynthesis	Amino Acid Metabolism
Cyanoamino acid metabolism	Metabolism of Other Amino Acids
Peptidoglycan biosynthesis	Glycan Biosynthesis and Metabolism
Histidine metabolism	Amino Acid Metabolism
Pentose phosphate pathway	Carbohydrate Metabolism
Ubiquinone and other terpenoid-quinone biosynthesis	Metabolism of Cofactors and Vitamins
Oxidative phosphorylation	Energy Metabolism

Alanine, aspartate and glutamate metabolism	Amino Acid Metabolism
Reductive carboxylate cycle (CO2 fixation)	Energy Metabolism
Folate biosynthesis	Metabolism of Cofactors and Vitamins
Fatty acid biosynthesis	Lipid Metabolism
Biotin metabolism	Metabolism of Cofactors and Vitamins
alpha-Linolenic acid metabolism	Lipid Metabolism
Geraniol degradation	Xenobiotics Biodegradation and Metabolism
Butanoate metabolism	Carbohydrate Metabolism
Fatty acid metabolism	Lipid Metabolism
Nicotinate and nicotinamide metabolism	Metabolism of Cofactors and Vitamins
Propanoate metabolism	Carbohydrate Metabolism
Citrate cycle (TCA cycle)	Carbohydrate Metabolism
D-Glutamine and D-glutamate metabolism	Metabolism of Other Amino Acids
Nitrogen metabolism	Energy Metabolism
Bisphenol A degradation	Xenobiotics Biodegradation and Metabolism
Porphyrin and chlorophyll metabolism	Metabolism of Cofactors and Vitamins
Pantothenate and CoA biosynthesis	Metabolism of Cofactors and Vitamins
Drug metabolism - other enzymes	Xenobiotics Biodegradation and Metabolism
1- and 2-Methylnaphthalene degradation	Xenobiotics Biodegradation and Metabolism
Ascorbate and aldarate metabolism	Carbohydrate Metabolism
Naphthalene and anthracene degradation	Xenobiotics Biodegradation and Metabolism
Amino sugar and nucleotide sugar metabolism	Carbohydrate Metabolism
Biosynthesis of siderophore group nonribosomal peptides	Biosynthesis of Polyketides and Nonribosomal Peptides
Riboflavin metabolism	Metabolism of Cofactors and Vitamins
One carbon pool by folate	Metabolism of Cofactors and Vitamins
Glycosphingolipid biosynthesis - globo series	Glycan Biosynthesis and Metabolism
High-mannose type N-glycan biosynthesis	Glycan Biosynthesis and Metabolism
Starch and sucrose metabolism	Carbohydrate Metabolism
Pyrimidine metabolism	Nucleotide Metabolism
Glycine, serine and threonine metabolism	Amino Acid Metabolism
Lysine biosynthesis	Amino Acid Metabolism
C5-Branched dibasic acid metabolism	Carbohydrate Metabolism
Methane metabolism	Energy Metabolism
Biphenyl degradation	Xenobiotics Biodegradation and Metabolism
Limonene and pinene degradation	Biosynthesis of Secondary Metabolites
Flavonoid biosynthesis	Biosynthesis of Secondary Metabolites

Tetracycline biosynthesis	Biosynthesis of Secondary Metabolites
Glyoxylate and dicarboxylate metabolism	Carbohydrate Metabolism
Caprolactam degradation	Xenobiotics Biodegradation and Metabolism
Valine, leucine and isoleucine degradation	Amino Acid Metabolism
Glycosphingolipid biosynthesis - ganglio series	Glycan Biosynthesis and Metabolism
Betalain biosynthesis	Biosynthesis of Secondary Metabolites
Biosynthesis of type II polyketide backbone	Biosynthesis of Polyketides and Nonribosomal Peptides
Selenoamino acid metabolism	Metabolism of Other Amino Acids
Sulfur metabolism	Energy Metabolism
Benzoate degradation via hydroxylation	Xenobiotics Biodegradation and Metabolism
Glycerophospholipid metabolism	Lipid Metabolism
Ether lipid metabolism	Lipid Metabolism
Diterpenoid biosynthesis	Biosynthesis of Secondary Metabolites
Ethylbenzene degradation	Xenobiotics Biodegradation and Metabolism
Lipoic acid metabolism	Metabolism of Cofactors and Vitamins
Lipopolysaccharide biosynthesis	Glycan Biosynthesis and Metabolism
Glycosylphosphatidylinositol(GPI)-anchor biosynthesis	Glycan Biosynthesis and Metabolism
Stilbenoid, diarylheptanoid and gingerol biosynthesis	Biosynthesis of Secondary Metabolites
Anthocyanin biosynthesis	Biosynthesis of Secondary Metabolites
Sphingolipid metabolism	Lipid Metabolism
O-Glycan biosynthesis	Glycan Biosynthesis and Metabolism
Glycerolipid metabolism	Lipid Metabolism
Biosynthesis of type II polyketide products	Biosynthesis of Polyketides and Nonribosomal Peptides
Streptomycin biosynthesis	Biosynthesis of Secondary Metabolites
Galactose metabolism	Carbohydrate Metabolism
Valine, leucine and isoleucine biosynthesis	Amino Acid Metabolism
Primary bile acid biosynthesis	Lipid Metabolism
Novobiocin biosynthesis	Biosynthesis of Secondary Metabolites
D-Alanine metabolism	Metabolism of Other Amino Acids
D-Arginine and D-ornithine metabolism	Metabolism of Other Amino Acids
Isoquinoline alkaloid biosynthesis	Biosynthesis of Secondary Metabolites
Glycosaminoglycan degradation	Glycan Biosynthesis and Metabolism
Taurine and hypotaurine metabolism	Metabolism of Other Amino Acids
Arachidonic acid metabolism	Lipid Metabolism
Biosynthesis of 12-, 14- and 16-membered macrolides	Biosynthesis of Polyketides and Nonribosomal Peptides

C21-Steroid hormone metabolism	Lipid Metabolism
Phosphonate and phosphinate metabolism	Metabolism of Other Amino Acids
Carotenoid biosynthesis	Biosynthesis of Secondary Metabolites
Photosynthesis	Energy Metabolism
Glycosphingolipid biosynthesis - lacto and neolacto series	Glycan Biosynthesis and Metabolism
Flavone and flavonol biosynthesis	Biosynthesis of Secondary Metabolites
Terpenoid backbone biosynthesis	Biosynthesis of Secondary Metabolites
Inositol phosphate metabolism	Carbohydrate Metabolism
Tetrachloroethene degradation	Xenobiotics Biodegradation and Metabolism
Biosynthesis of ansamycins	Biosynthesis of Polyketides and Nonribosomal Peptides
Penicillin and cephalosporin biosynthesis	Biosynthesis of Secondary Metabolites
2,4-Dichlorobenzoate degradation	Xenobiotics Biodegradation and Metabolism
beta-Lactam resistance	Biosynthesis of Secondary Metabolites
Biosynthesis of vancomycin group antibiotics	Biosynthesis of Polyketides and Nonribosomal Peptides
Polyketide sugar unit biosynthesis	Biosynthesis of Polyketides and Nonribosomal Peptides
Styrene degradation	Xenobiotics Biodegradation and Metabolism
Fatty acid elongation in mitochondria	Lipid Metabolism
Phosphatidylinositol signaling system	Signal Transduction
Zeatin biosynthesis	Biosynthesis of Secondary Metabolites
T cell receptor signaling pathway	Immune System
Linoleic acid metabolism	Lipid Metabolism
Secondary bile acid biosynthesis	Lipid Metabolism
Retinol metabolism	Metabolism of Cofactors and Vitamins
Atrazine degradation	Xenobiotics Biodegradation and Metabolism
Biosynthesis of unsaturated fatty acids	Lipid Metabolism

Supplementary Table 5: List of all the beta lactamase genes present in publicly available 86 genomes of *P. stuartii*

Source	Genome Name	Gene	Identity	E-value
CARD	Providencia stuartii strain 2021EL-01179	OXA-9	100	1.00E-160
CARD	Providencia stuartii TUM3764	CTX-M-2	100	1.00E-162
CARD	Providencia stuartii AUH_PS_2022_1	OXA-10	100	1.00E-151
CARD	Providencia stuartii CAVP496	OXA-9	100	1.00E-160
CARD	Providencia stuartii strain Ps850	TEM-84	100	1.00E-143
CARD	Providencia stuartii strain PRV00021	TEM-1	100	1.00E-163
CARD	Providencia stuartii strain PS901	TEM-12	100	8.00E-11
CARD	Providencia stuartii strain 3347685	OXA-1	100	1.00E-158
CARD	Providencia stuartii strain 2021EL-01138	OXA-9	100	1.00E-160
CARD	Providencia stuartii CMC-4104	NDM-1	100	1.00E-156
CARD	Providencia stuartii TUM13246	TEM-1	100	1.00E-163
CARD	Providencia stuartii strain 4595 strain not applicable	OXA-1	100	1.00E-158
CARD	Providencia stuartii strain 2020EL-00004	IMP-27	100	1.00E-144
CARD	Providencia stuartii strain M2	NDM-1	100	1.00E-156
CARD	Providencia stuartii strain NCTC12257	TEM-2	100	1.00E-163
CARD	Providencia stuartii CAVP450	PER-1	100	1.00E-172
CARD	Providencia stuartii CAVP450	OXA-10	100	1.00E-151
CARD	Providencia stuartii strain NCTC12257	TEM-2	100	1.00E-163
CARD	Providencia stuartii strain 2021EL-01179	CTX-M-15	100	1.00E-161
CARD	Providencia stuartii CAVP450	OXA-9	100	1.00E-160

CARD	Providencia stuartii strain BML2537	IMP-10	99	1.00E-142
CARD	Providencia stuartii strain PS901	OXA-1	100	1.00E-158
CARD	Providencia stuartii strain 2021EL-01138	CTX-M-15	100	1.00E-161
CARD	Providencia stuartii CMC-4104	PER-1	100	1.00E-172
CARD	Providencia stuartii TUM13246	CTX-M-14	100	1.00E-163
CARD	Providencia stuartii CAVP496	TEM-1	100	1.00E-163
CARD	Providencia stuartii CCBH26698	NDM-1	100	1.00E-156
CARD	Providencia stuartii 2021CK-01196	IMP-27	100	1.00E-144
CARD	Providencia stuartii CAVP496	PER-1	100	1.00E-172
CARD	Providencia stuartii D5720	DHA-1	100	1.00E-219
CARD	Providencia stuartii strain M5	OXA-10	100	1.00E-151
CARD	Providencia stuartii strain NCTC10835	TEM-2	100	1.00E-163
CARD	Providencia stuartii strain F3W	NDM-1	100	1.00E-156
CARD	Providencia stuartii strain 2021EL-01138	TEM-1	100	1.00E-163
CARD	Providencia stuartii 2021CK-01196	TEM-1	100	1.00E-163
CARD	Providencia stuartii CMC-4104	OXA-10	100	1.00E-151
CARD	Providencia stuartii strain AS012498	SHV-7	100	1.00E-78
CARD	Providencia stuartii strain F3W	OXA-10	100	1.00E-151
CARD	Providencia stuartii TUM13246	CTX-M-14	100	1.00E-163
CARD	Providencia stuartii CAVP450	TEM-1	100	1.00E-163
CARD	Providencia stuartii P14	OXA-10	100	1.00E-151
CARD	Providencia stuartii SHNIBPS63	VEB-6	100	1.00E-171
CARD	Providencia stuartii strain PRV00022	TEM-1	100	1.00E-163

CARD	Providencia stuartii strain Ps848	OXA-1	100	1.00E-158
CARD	Providencia stuartii strain FDAARGOS_645 strain Not applicable	OXA-10	100	1.00E-151
CARD	Providencia stuartii strain PS901	TEM-93	100	1.00E-145
CARD	Providencia stuartii strain MF1	DHA-1	100	1.00E-219
CARD	Providencia stuartii strain Ps848	NDM-1	100	1.00E-156
CARD	Providencia stuartii strain NCTC12257	TEM-2	100	1.00E-163
CARD	Providencia stuartii strain 3347685	CTX-M-15	100	1.00E-161
CARD	Providencia stuartii strain PRV00021	OXA-2	100	1.00E-160
CARD	Providencia stuartii CAVP496	OXA-10	100	1.00E-151
CARD	Providencia stuartii CCBH26698	CTX-M-8	100	1.00E-162
CARD	Providencia stuartii CAVP490	TEM-1	100	1.00E-163
CARD	Providencia stuartii CAVP490	OXA-9	100	1.00E-160
CARD	Providencia stuartii strain 3347685	OXA-48	100	1.00E-154
CARD	Providencia stuartii 2021CK-01296	IMP-27	100	1.00E-144
CARD	Providencia stuartii TUM13241	CTX-M-2	100	1.00E-162
CARD	Providencia stuartii AUH_PS_2022_1	NDM-1	100	1.00E-146
CARD	Providencia stuartii strain 2021EL-01179	OXA-1	100	1.00E-158
CARD	Providencia stuartii strain 2021EL-01179	KPC-3	100	1.00E-167
CARD	Providencia stuartii SHNIBPS63	OXA-10	100	1.00E-151
CARD	Providencia stuartii strain PRV00022	CTX-M-2	100	1.00E-162
CARD	Providencia stuartii CAVP496	SHV-7	100	1.00E-135
CARD	Providencia stuartii CAVP496	NDM-1	100	1.00E-156
CARD	Providencia stuartii CAVP490	NDM-1	100	1.00E-156

CARD	Providencia stuartii SHNIBPS63	NDM-1	100	1.00E-156
CARD	Providencia stuartii strain 3347685	TEM-1	100	1.00E-163
CARD	Providencia stuartii strain Ps850	OXA-1	100	1.00E-158
CARD	Providencia stuartii CAVP490	SHV-7	100	1.00E-135
CARD	Providencia stuartii strain 2021EL-01138	OXA-1	100	1.00E-158
CARD	Providencia stuartii strain 2021EL-01138	KPC-3	100	1.00E-167
CARD	Providencia stuartii strain 3347685	CMY-16	100	1.00E-226
CARD	Providencia stuartii strain Ps850	NDM-1	100	1.00E-156
CARD	Providencia stuartii strain M2	OXA-10	100	1.00E-151
CARD	Providencia stuartii strain M4	OXA-10	100	1.00E-151
CARD	Providencia stuartii strain AS012498	SHV-13	100	2.00E-79
CARD	Providencia stuartii SHNIBPS71	CMY-16	100	1.00E-226
CARD	Providencia stuartii TUM3764	CTX-M-2	100	1.00E-162
CARD	Providencia stuartii strain 3347685	OXA-10	100	1.00E-151
CARD	Providencia stuartii TUM13246	TEM-1	100	1.00E-163
CARD	Providencia stuartii strain 4595 strain not applicable	CTX-M-15	100	1.00E-161
CARD	Providencia stuartii CMC-4104	PER-1	100	1.00E-172
CARD	Providencia stuartii strain M4	NDM-1	100	1.00E-156
CARD	Providencia stuartii strain PRV00022	OXA-2	100	1.00E-160
CARD	Providencia stuartii strain PRV00021	CTX-M-2	100	1.00E-162
CARD	Providencia stuartii CAVP490	OXA-10	100	1.00E-151
CARD	Providencia stuartii strain PRV00003	TEM-1	100	1.00E-163
CARD	Providencia stuartii 2020CK-00448	TEM-141	99	1.00E-104

CARD	Providencia stuartii strain 2021EL-01179	TEM-1	100	1.00E-163
CARD	Providencia stuartii CAVP450	NDM-1	100	1.00E-156
CARD	Providencia stuartii strain 2020EL-00072	IMP-27	100	1.00E-144
CARD	Providencia stuartii strain PS901	NDM-1	100	1.00E-156
CARD	Providencia stuartii strain 3347685	CTX-M-15	100	1.00E-161
CARD	Providencia stuartii TUM13241	CTX-M-2	100	1.00E-162
CARD	Providencia stuartii CCBH26698	OXA-10	100	1.00E-151
CARD	Providencia stuartii CAVP450	SHV-7	100	1.00E-135
CARD	Providencia stuartii strain M5	NDM-1	100	1.00E-156
CARD	Providencia stuartii 2020CK-00448	KPC-2	100	1.00E-167
CARD	Providencia stuartii AUH_PS_2022_1	CMY-12	100	1.00E-192
CARD	Providencia stuartii P14	CMY-2	100	1.00E-227
CARD	Providencia stuartii strain Ps848	TEM-84	100	1.00E-143

Supplementary Table 6: Result of Pangenome analysis

Criteria	Number of Genes
Core genes (99% <= strains <= 100%)	195
Soft core genes (95% <= strains < 99%)	165
Shell genes (15% <= strains < 95%)	4,392
Cloud genes (0% <= strains < 15%)	23,066
Total genes (0% <= strains <= 100%)	27,818
